# MARCO promotes cholangiocarcinogenesis by inducing immunosuppression and its targeting reduces tumor growth

**DOI:** 10.1038/s41392-026-02657-w

**Published:** 2026-04-29

**Authors:** Aloña Agirre-Lizaso, Maider Huici-Izagirre, Colm J. O’Rourke, Ekaterina Zhuravleva, Guido Carpino, Diletta Overi, Josu Urretabizkaia-Garmendia, Ibone Labiano, Ana Korosec, Beatriz Val, Diego F. Calvisi, Sumera I. Ilyas, Joke M. M. den Haan, Patricia Aspichueta, Elizabeth Hijona, Adelaida La Casta, Raúl Jiménez-Agüero, Ana Lleo, Rocio I. R. Macias, Eugenio Gaudio, European CCA Histology Registry, Jesper B. Andersen, Gernot Schabbauer, Luis Bujanda, Pedro M. Rodrigues, Omar Sharif, Jesus M. Banales, Maria J. Perugorria

**Affiliations:** 1 Department of Liver and Gastrointestinal Diseases, Biogipuzkoa Health Research Institute - Donostia University Hospital -, University of the Basque Country (UPV/EHU), San Sebastian, Spain; 2 Biotech Research and Innovation Centre (BRIC), Department of Health and Medical Sciences, University of Copenhagen, Copenhagen, Denmark; 3 Department of Anatomical, Histological, Forensic Medicine and Orthopedic Sciences, Sapienza University of Rome, Rome, Italy; 4 Translational Medical Oncology Research Unit, Navarrabiomed-Instituto de Investigación Sanitaria de Navarra (IdiSNA), Pamplona, Spain; 5 Institute for Vascular Biology, Centre for Physiology and Pharmacology, Medical University Vienna, Vienna, Austria; 6 Christian Doppler Laboratory for Immunometabolism and Systems Biology of Obesity-Related Diseases (InSpiReD), Vienna, Austria; 7 CIBERehd, Instituto de Salud Carlos III (ISCIII), Madrid, Spain; 8 Institute of Pathology, University of Regensburg, Regensburg, Germany; 9 Division of Gastroenterology and Hepatology, Mayo Clinic, Rochester, MN USA; 10 Department of Molecular Cell Biology and Immunology, Cancer Center Amsterdam, Amsterdam Infection and Immunity Institute, Amsterdam UMC, Vrije Universiteit Amsterdam, Amsterdam, The Netherlands; 11 Department of Physiology, Faculty of Medicine and Nursing, University of the Basque Country (UPV/EHU), Leioa, Spain; 12 Biobizkaia Health Research Institute, Barakaldo, Spain; 13 Department of Biomedical Sciences, Humanitas University, Milan, Italy; 14 Division of Internal Medicine and Hepatology, Department of Gastroenterology, IRCCS Humanitas Research Hospital, Rozzano, Milan, Italy; 15 Laboratory of Experimental Hepatology and Drug Targeting (HEVEPHARM), IBSAL, University of Salamanca, Salamanca, Spain; 16 Christian Doppler Laboratory for Arginine Metabolism in Rheumatoid Arthritis and Multiple Sclerosis, Vienna, Austria; 17 IKERBASQUE, Basque Foundation for Science, Bilbao, Spain; 18 Department of Biochemistry and Genetics, School of Sciences, University of Navarra, Pamplona, Spain; 19 Department of Medicine, Faculty of Medicine and Nursing, University of the Basque Country (UPV/EHU), San Sebastián, Spain

**Keywords:** Gastrointestinal cancer, Cancer microenvironment

## Abstract

Cholangiocarcinoma (CCA) comprises a heterogeneous group of biliary malignant tumors with poor prognosis and limited therapeutic options. Recent studies have highlighted the role of the immune system in the development and progression of intrahepatic CCA (iCCA). In this study, we investigated the role of the scavenger receptor MARCO in iCCA. Employing transcriptomic, spatial proteomic and histological analyses of human samples, MARCO was found on a specific subtype of tumor-associated macrophages linked to immunosuppression and extracellular matrix remodeling within the tumor microenvironment. High MARCO expression in human iCCA tumors correlated with worse overall survival, T cell dysfunction and increased collagen deposition. In line with this, MARCO expression was associated with a T_H_2-skewed immune response and was increased in macrophages exposed to IL-4 and IL-13. *Marco*^*−/−*^ mice were protected against iCCA development, exhibiting reduced tumor burden, fewer innate immune cells related to T_H_2 responses and attenuated fibrosis. Moreover, *Marco*^*−/−*^ mice exhibited lower levels of immunosuppressive markers on macrophages and cytotoxic T cells, resulting in improved overall survival and reduced lung metastases in an orthotopic tumor model. The use of an anti-MARCO antibody further reduced tumor volume in wild-type mice. This study identifies MARCO as a key regulator of immunosuppression, fibrosis and tumor progression in iCCA, and supports its potential as a novel therapeutic target for macrophage-directed immunotherapy.

## Introduction

Cholangiocarcinoma (CCA) comprises a diverse group of biliary cancers classified by the World Health Organization (WHO) in 2019 into intrahepatic CCA (iCCA), perihilar CCA (pCCA) or distal CCA (dCCA), based on differences in etiology, pathogenesis, incidence, and prognosis.^1,2^ It is the second most common primary liver cancer and its global incidence continues to increase. CCA often presents asymptomatically in early stages, and is usually diagnosed at an advanced stage, making curative surgery unfeasible.^3^ Furthermore, recurrence occurs in approximately 70% of cases post-surgery.^4^ The current first-line therapy for patients with advanced disease, consisting of gemcitabine and cisplatin combined with immune checkpoint inhibitors (such as durvalumab or pembrolizumab),^5,6^ is mainly palliative. Consequently, survival rates are low, with only 7–20% of patients surviving 5 years, and an average survival of approximately 6 months after diagnosis.^1,3,7^ This highlights the urgent need for better understanding of CCA biology to develop more effective treatments.

Recent advances in tumor immunobiology have highlighted the critical role of the tumor microenvironment (TME) in cancer progression and therapeutic resistance. Classification of iCCAs according to their TME has revealed that more than 60% of tumors present a non-inflamed profile, characterized by the absence of CD8^+^ T cells and a predominance of immunosuppressive populations, including M2-type tumor-associated macrophages (TAMs) and myeloid-derived suppressor cells (MDSCs).^8^ In addition to promoting an immunosuppressive microenvironment, TAMs interact closely with extracellular matrix (ECM) components and fibroblasts, contributing to stromal remodeling, further restricting immune cell infiltration, and reducing the efficacy of therapies. These findings suggest that selective targeting of TAMs could offer therapeutic benefit in iCCA. Indeed, TAMs are highly abundant within the TME and therefore, several therapeutic strategies are being investigated, including TAM depletion, inhibition of TAM recruitment, regulation of phagocytosis by TAMs, and reprogramming of macrophages to enhance their anti-tumor capacity.^9,10^ Nevertheless, in iCCA, well-defined macrophage-associated targets remain poorly characterized, limiting the successful translation of immunomodulatory strategies into clinical interventions.

The scavenger receptor MARCO was recently found to be expressed in a subtype of TAMs with an M2-like immunosuppressive signature, correlating with poor prognosis in several human solid cancers.^11–14^ MARCO plays a key role in macrophage polarization and consequently in adaptive immune responses.^15^ In the liver, a novel subset of immunosuppressive MARCO-expressing resident macrophages was characterized. This subset expresses high levels of interleukin-10 and preferentially localizes in the periportal vein zone.^16^ In addition, MARCO was described to regulate tissue fibrosis in the lungs.^17^ Notably, antibody-mediated targeting MARCO-expressing TAMs was shown to block tumor growth and metastasis in breast cancer and melanoma through reprogramming TAM populations to a pro-inflammatory phenotype and increasing tumor immunogenicity in experimental models in vivo.^15^ Despite these advances, the functional role of MARCO in iCCA and the nature of MARCO-expressing cells within the iCCA TME remain largely unexplored.

Here, we aimed to elucidate the role and therapeutic potential of MARCO in iCCA. Using integrated transcriptomic, spatial proteomic and histological analyses of human iCCA samples, we identified MARCO as a marker of a subset of TAMs associated with immunosuppression, ECM remodeling, tumor dissemination and poor patient outcomes. Our findings indicate that MARCO expression is driven by a T_H_2-skewed microenvironment and that MARCO^+^ TAMs have diverse origins, as they express markers of both Kupffer cells and monocyte-derived macrophages. Spatial proteomics analyses further demonstrated that MARCO^+^ TAMs were enriched in areas with high collagen deposition in iCCA. Using multiple mouse models of iCCA, we demonstrate that loss of MARCO attenuates tumor development, reduces fibrosis, limits metastatic dissemination and improves overall survival, accompanied by broad changes in the immune microenvironment. Notably, *Marco*^*−/−*^ mice exhibited reductions in immunosuppressive macrophages and T cell populations, along with diminished T_H_2-associated immune populations. Finally, we provide evidence that therapeutic targeting of MARCO reduces tumor growth in vivo. Together, these findings establish MARCO as a key regulator of the iCCA tumor microenvironment and a promising target for macrophage-directed immunotherapy.

## Results

### *MARCO* is expressed in TAMs in human iCCA tumors, correlating with worse overall survival (OS)

**Fig. 1:**
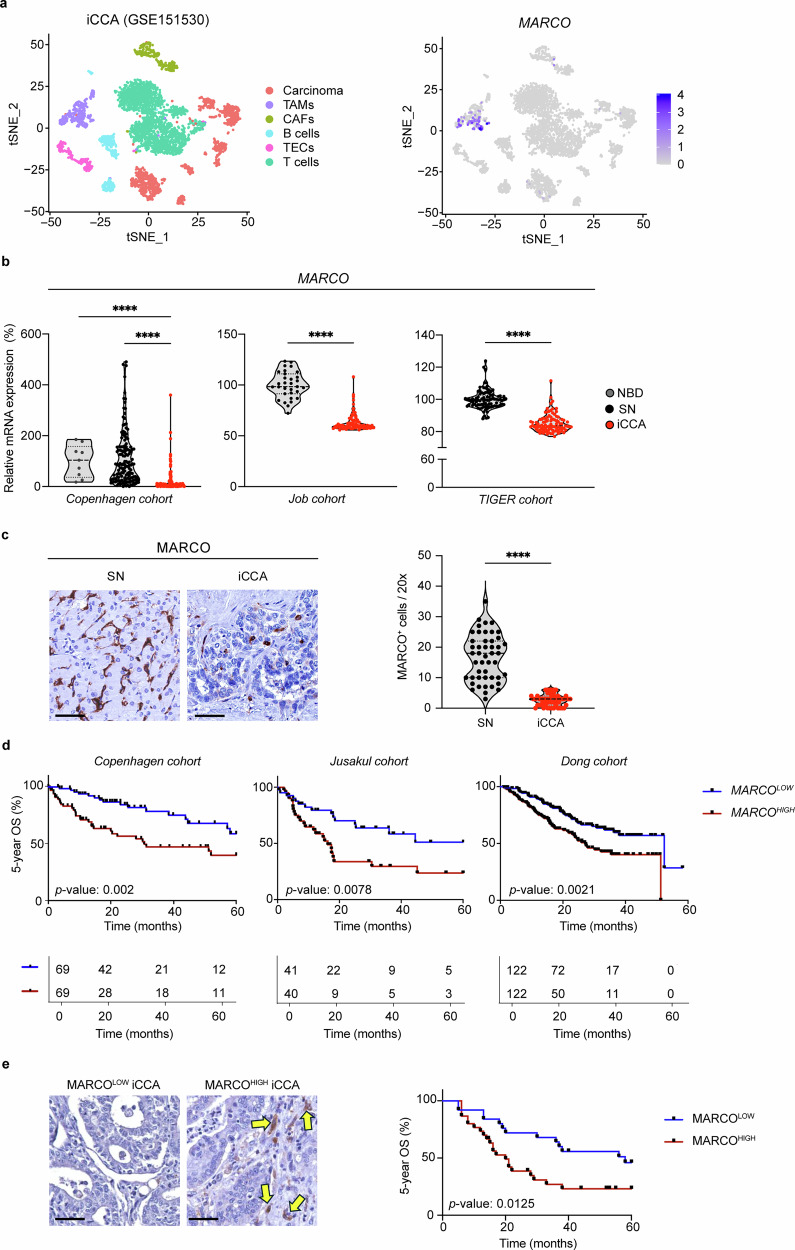
*MARCO* is expressed in tumor-associated macrophages (TAMs) and correlates with poor patient survival. **a** Left: graph-based clustering using t-distributed stochastic neighbour embedding (t-SNE) projection depicting the cellular composition of different human samples. Cells that share similar transcriptome profiles are grouped by colors and were annotated using lineage specific markers. Right: t-SNE plot depicting *MARCO* expression in TAMs in iCCA tumors [GSE151530 (*n* = 12)]. **b** *MARCO* mRNA (microarray) expression in iCCA tumors compared to surrounding normal (SN) and normal bile ducts (NBD) in the Copenhagen cohort (iCCA, *n* = 151; SN, *n* = 143; NBD, *n* = 9), Job cohort (iCCA, *n* = 78; SN, *n* = 31) and TIGER cohort (iCCA, *n* = 91; SN, *n* = 91). Data are shown as mean ± SEM. Wilcoxon test was used. **** represents a *P* value of <0.0001. **c** MARCO protein expression in iCCA tumors compared to SN in samples from the European CCA Histology Registry (iCCA, *n* = 55; SN, *n* = 47). Scale bar = 50 µm. Data are shown as mean ± SEM. **** represents a *P* value of <0.0001. **d** Five-year OS curves of patients with iCCA from the Copenhagen (*n* = 120), Jusakul (*n* = 81) and Dong (*n* = 244) cohorts. Red and blue lines indicate patients with iCCA classified according to high *MARCO* (above median) *vs* low *MARCO* (at or below median) expression, respectively. Log-rank test was used. **e** Immunohistochemistry for MARCO on iCCA samples. Tumors were dichotomized into MARCO^LOW^ and MARCO^HIGH^ iCCA based on the median number of MARCO^+^ cells per high-powered microscopic field. Representative images are shown; arrows indicate MARCO^+^ cells. The 5-year OS curves of patients with iCCA from the European CCA Histology Registry (*n* = 55) are shown on the right. Log-rank test was performed. CAF cancer-associated fibroblast, iCCA intrahepatic cholangiocarcinoma, MARCO macrophage receptor with collagenous structure, NBD normal bile duct, SN surrounding normal, TAM tumor-associated macrophage, TEC tumor endothelial cell, tSNE t-distributed stochastic neighbour embedding

To investigate the potential role of MARCO in human iCCA, we first analyzed *MARCO* expression in healthy and cirrhotic human livers, as well as in iCCA tumors, utilizing publicly available single-cell RNA sequencing data. *MARCO* is mainly expressed in macrophages in normal and cirrhotic livers, and not in other non-parenchymal or liver epithelial cells (Supplementary Fig. 1a, b). In iCCA tumors, the expression of *MARCO* was restricted to TAMs (Fig. 1a and Supplementary Fig. 1c, d). Immunofluorescence confirmed this finding, additionally demonstrating MARCO expression within a subset of CD68^+^ or CD206^+^ TAMs (Supplementary Fig. 2a).

*MARCO* expression levels were lower in human iCCA samples compared to non-tumor liver tissues [non-tumor surrounding normal (SN) and normal bile ducts (NBD)] in three independent patient cohorts^18–21^ (Fig. 1b). Of note, while *MARCO* expression was lower in all iCCA tumors regardless of their mutational profile, compared to NBD or SN liver tissues, *KRAS* mutated tumors and advanced-stage (Stage IV) tumors exhibited the highest *MARCO* expression (Supplementary Fig 2b, c). To strengthen these findings, we next examined MARCO protein levels in 55 iCCA human tissue samples from the ENS-CCA Histological Registry. Immunohistochemistry confirmed higher MARCO expression levels in the surrounding liver tissue compared to the tumor, although some tumors also exhibited MARCO expression within the tumor (Fig. 1c).

We next addressed the potential prognostic value of *MARCO* expression in iCCA tumors. Importantly, in three independent cohorts,^18,22,23^ high *MARCO* tumor expression (above the median) was associated with worse patients’ OS (Fig. 1d). This was independent of the presence of *KRAS* mutations in these tumors, and both *MARCO* expression and *KRAS* mutations were found to be independent markers of poor prognosis (Supplementary Fig. 2d). At the protein level, classifying samples from the ENS-CCA Histological Registry as MARCO^LOW^ or MARCO^HIGH^ based on the median value of the number of MARCO^+^ cells within the tumor mass demonstrated that MARCO^HIGH^ iCCAs exhibited a worse 5-year OS than MARCO^LOW^ iCCAs, confirming previous observations at the RNA level (Fig. 1e).

### *MARCO*^*+*^ TAMs are involved in immunosuppression and ECM remodeling

**Fig. 2:**
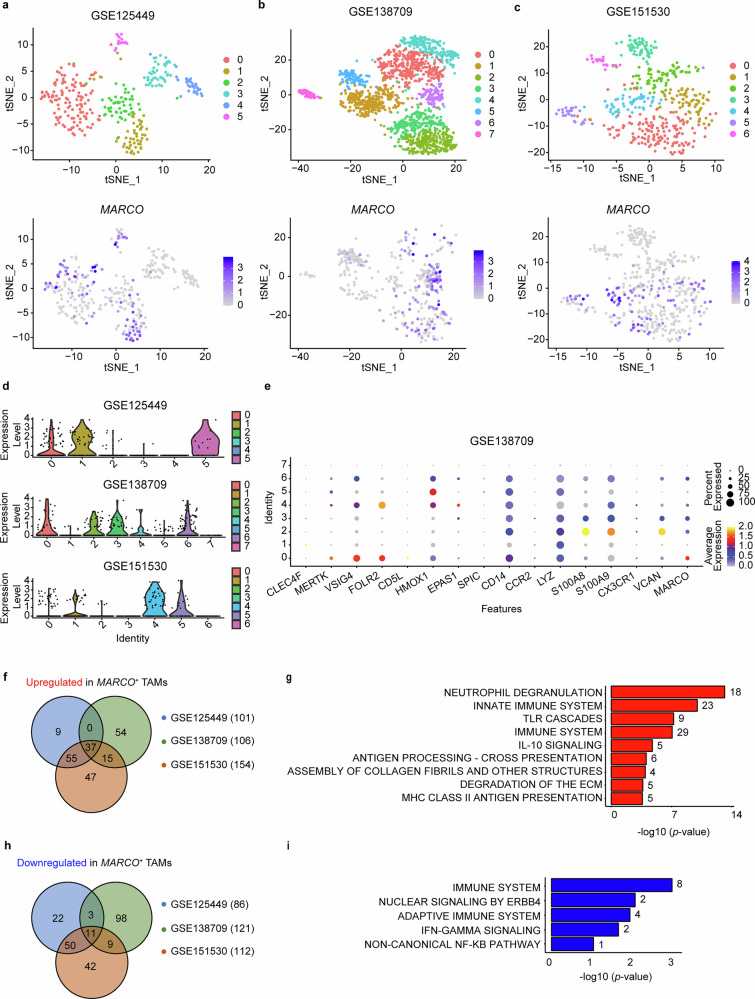
Analysis of *MARCO* expression in TAMs by single-cell RNA sequencing. **a**–**c** Graph-based clustering using t-distributed stochastic neighbour embedding (t-SNE) projection depicting different TAM compositions in three independent human iCCA datasets. TAMs that share similar transcriptome profiles are clustered by numbers and *MARCO* expression is depicted therein **a** GSE125449 (*n* = 10), **b** GSE138709 (*n* = 4 T, 3 SL) and **c** GSE151530 (*n* = 12). **d** Violin plots illustrating the distribution of *MARCO* expression across the identified TAM cluster. **e** Expression of key KC and monocytic markers in the identified TAM clusters in GSE138709. **f** Venn diagram including genes that are increased in *MARCO*^*+*^ *vs MARCO*^*-*^ TAMs in the three cohorts of patients with iCCA. **g** Reactome analysis of the commonly upregulated genes. **h** Venn diagram including genes that have decreased expression in *MARCO*^*+*^ *vs MARCO*^*-*^ TAMs. **i** Reactome analysis of the commonly downregulated genes. MARCO macrophage receptor with collagenous structure, TAM tumor-associated macrophage, tSNE t-distributed stochastic neighbour embedding

We next characterized *MARCO-*expressing (*MARCO*^*+*^) TAMs. The refined clustering of TAMs in three independent iCCA patient cohorts revealed distinct clusters with prominent *MARCO* expression (Fig. 2a–d). Suggesting a heterogeneous origin, *MARCO*-expressing TAMs expressed markers of both Kupffer cells (KCs) (*VSIG4, FOLR2, HMOX1*) and monocyte-derived macrophages (*CD14, LYZ, S100A8, S100A9, VCAN)* (Fig. 2e and Supplementary Fig. 3a).^24–27^ Comparing *MARCO*^*+*^ and *MARCO*^*-*^ TAMs revealed differential gene expression profiles (Supplementary Fig. 3b). Reactome analysis of genes commonly upregulated in *MARCO*^*+*^ TAMs indicated involvement of pathways related to neutrophil degranulation (e.g., *S100A8, S100A9, LGALS3*) which is linked to immunosuppression in other solid carcinomas^28^ or anti-inflammatory and immunosuppressive interleukin-10 (IL-10) signaling.^29^ Suggestive of a role in fibrosis, *MARCO*^*+*^ TAMs were also associated with pathways related to the assembly of collagen fibrils and the degradation of ECM (e.g., *CTSB, CTSD, CTSL, CTSSS, VCAN*) (Fig. 2f, g). Conversely, gene sets downregulated in *MARCO*^*+*^ TAMs were associated with the immune system and interferon gamma signaling (Fig. 2h, i).

Noting the heterogeneous nature of *MARCO* expressing TAMs (Fig. 2e and Supplementary Fig. 3a), we next compared *MARCO*^*+*^ TAMs in the TME with *MARCO*^+^ macrophages in non-tumor sites (healthy or SN liver) in all aforementioned datasets (Supplementary Fig. 4a–d). These data indicated that compared to *MARCO*^*+*^ macrophages in non-tumor tissue, *MARCO*^*+*^ TAMs lose key homeostatic and immunological functions typically attributed to KCs such as complement cascade activity, heme scavenging and binding/uptake of ligands by scavenger receptors.^30–32^ In this analysis, we also noted that gene sets upregulated in *MARCO*^+^ TAMs in comparison to *MARCO*^+^ macrophages in non-tumor sites (healthy or SN liver) included those related to mitosis, cell cycle and collagen chain trimerization (Supplementary Fig. 4e). Together, these findings suggest that *MARCO* expression within TAMs may impact iCCA development and progression, possibly via immunosuppression and ECM remodeling. We thus next correlated *MARCO* expression with all the mRNA transcripts expressed in iCCA tumors from different cohorts of patients. Reactome analysis of the common genes positively correlating with *MARCO* ratified that *MARCO* expression in iCCA samples was associated with neutrophil degranulation, IL-10 signaling, collagen biosynthesis/modification and notably IL-4/IL-13 signaling (Supplementary Fig. 5a). Genes negatively correlating with *MARCO* expression were involved in FGFR and PI3K signaling pathways, insulin receptor signaling, and the assembly of the cilium (Supplementary Fig. 5b), a sensory organelle key for cholangiocyte differentiation.^33^ These results suggest that MARCO regulates signaling pathways involved in cholangiocarcinogenesis and that type 2 cytokines found in the TME might promote *MARCO* expression within TAMs. Supportive, exogenous treatment of macrophages with IL-4/IL-13 robustly induced *MARCO* expression (Supplementary Fig. 5c, d). Noting this, we next determined which cell types in the SN tissue and tumor of iCCA patients produce IL-4 and IL-13. Suggesting that in the context of iCCA a subset of T cells likely polarized to T_H_2 cells augment *MARCO* expression, scRNA-seq analysis revealed T cells exclusively produce IL-4/IL-13 (Supplementary Fig. 6).

### *MARCO* expression is associated with T_H_2 responses and immunosuppression in iCCA

Considering the association of *MARCO*^*+*^ TAMs with immune responses, we next used the Consensus^TME^ tool,^34^ correlating *MARCO* expression with 18 innate and adaptive immune cell gene signatures in iCCA samples.^22^ *MARCO* expression was most strongly associated with immune cell types involved in tumor progression, such as M2 macrophages and fibroblasts, and not with other cell types that present a direct anti-tumoral response, such as NK cells (Supplementary Fig. 7).

**Fig. 3:**
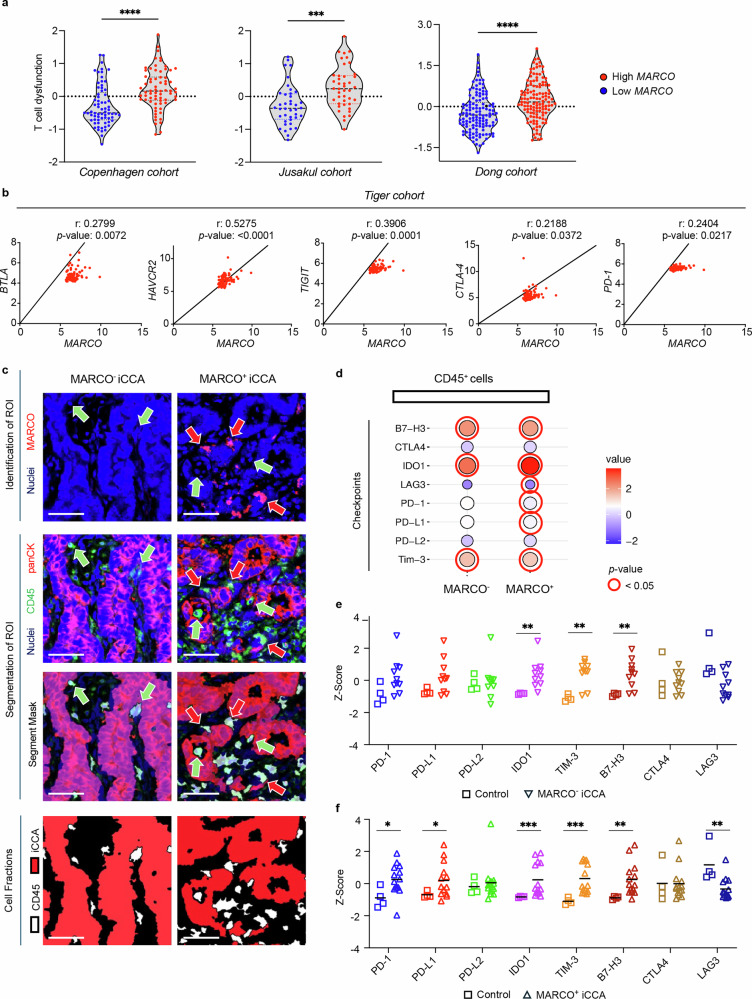
*MARCO* expression is associated with T cell exhaustion and dysfunction in iCCA. **a** T cell dysfunction score in human iCCA tumors according to *MARCO* expression employing the TIDE algorithm. Samples were categorized into low or high *MARCO* expression groups based on the median of *MARCO* mRNA expression levels in the Copenhagen (*n* = 138), Jusakul (*n* = 82) and Dong (*n* = 244) cohorts of patients. Data are shown as mean ± SEM. *** and **** represent *P* values of <0.001 and <0.0001, respectively. **b** Correlation of *MARCO* mRNA expression with genes linked to T cell exhaustion in iCCA tumors from the TIGER cohort of patients (*n* = 91). Parametric Pearson and non-parametric Spearman’s rank correlation test were used, correlation coefficient (r) and *P* value are shown. **c** Spatial proteomic analysis comparing MARCO^+^ and MARCO^−^ regions within iCCA tumors, based on the presence or absence of MARCO^+^ cells identified by immunofluorescence. CD45^+^ immune cells and tumor epithelial cell fractions were segmented and analyzed separately. Green arrows indicate some CD45^+^ cells, whereas red arrows highlight MARCO^+^ cells. Scale bar = 50 µm. **d** Comparison of the CD45^+^ immune cell fraction in iCCA tissue with CD45^+^ cells from normal portal tracts, categorized by MARCO^+^ and MARCO^-^ regions. Red circle indicates significance versus normal portal tracts. **e**, **f** Dot plots showing immune checkpoint protein expression in the CD45^+^ cell fraction as sampled by spatial proteomics analysis in **e** MARCO^−^ and **f** MARCO^+^ areas compared to normal portal tracts (controls). Individual values and means are shown. Mann–Whitney test was used. *, ** and *** denote *P* values of <0.05, <0.01 and <0.001, respectively. B7-H3 B7 homolog 3, BTLA B and T lymphocyte associated, CTLA-4 cytotoxic T-lymphocyte-associated protein 4, HAVCR2 hepatitis A virus cellular receptor 2, IDO1 Indoleamine 2,3-dioxygenase 1, LAG3 lymphocyte activation gene 3, MARCO Macrophage receptor with collagenous structure, panCK pan-cytokeratin, PD-1 programmed cell death protein 1, PD-L1 programmed Death-Ligand 1, TIGIT T cell immunoreceptor with Ig and ITIM domains, TIM3 T-cell immunoglobulin and mucin-domain containing-3

Using the tumor immune dysfunction and exclusion (TIDE) algorithm,^35^ high *MARCO*-expressing tumors were found to exhibit greater T cell dysfunctionality than low *MARCO*-expressing tumors in three independent patient cohorts (Fig. 3a). Similarly, specific correlations of *MARCO* with markers of T cell exhaustion revealed *MARCO* positively correlated with markers of T cell exhaustion (Fig. 3b).

To confirm these observations, we next performed a spatial proteomic analysis on an independent cohort of iCCA samples, identifying MARCO^+^ and MARCO^−^ areas using immunofluorescence. In this setting, CD45^+^ immune cells and tumor epithelial cell fractions were segmented and analyzed separately (Fig. 3c). When comparing CD45^+^ immune cells within tumors to those in normal portal tracts, we observed a profound upregulation of several immunosuppressive markers. Notably, MARCO^+^ tumors exhibited elevated PD-L1 and PD-1 protein expression, suggesting MARCO-expressing TAMs modulate PD-L1/PD-1 signaling in the iCCA TME (Fig. 3d–f).

**Fig. 4:**
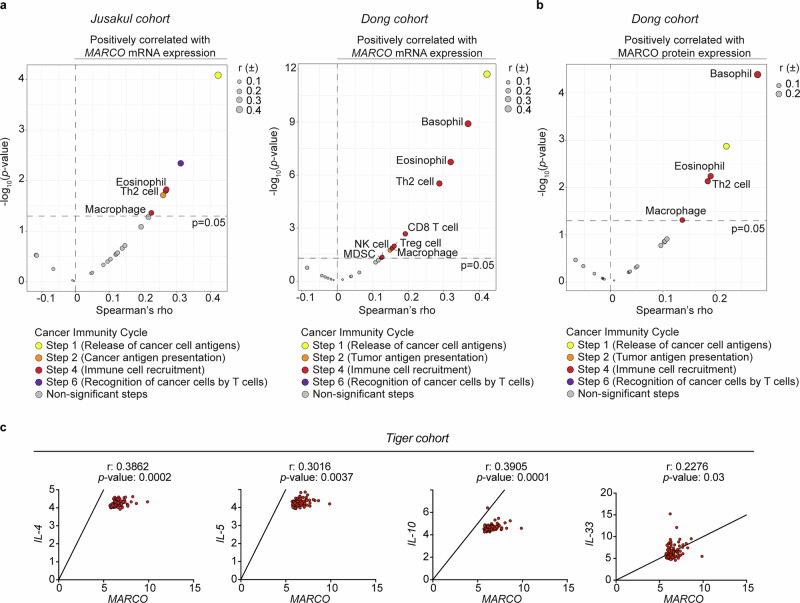
*MARCO* expression is associated with the T_H_2 response. **a** Volcano plot illustrating the correlation between *MARCO* mRNA expression and different stages of the cancer-immunity cycle in iCCA samples from the Jusakul (*n* = 82) and Dong (*n* = 244) cohorts of patients. Spearman’s rank correlation coefficient was used. **b** Volcano plot depicting the correlation of MARCO protein expression with different stages of the cancer-immunity cycle in iCCA samples from the Dong (*n* = 208) cohort of patients. Spearman’s rank correlation coefficient was used. **c** Correlation of *MARCO* mRNA expression with genes linked to a T_H_2 response in iCCA tumors from the TIGER cohort of patients (*n* = 91). Spearman’s rank correlation coefficient was used, correlation coefficient (r) and *P* value are shown. IL interleukin, MARCO macrophage receptor with collagenous structure

We next explored the broader immunosuppressive phenotype associated with MARCO expression. Therein, the tumor immunophenotype profiling (TIP) tool^36^ was used to assess immune activity during the cancer-immunity cycle. *MARCO* expression was positively associated with several defined steps of the tumor-immunity cycle, including the release of cancer cell antigens (step 1), presentation of tumor antigens (step 2), traffic or recruitment of immune cells to the tumor, such as eosinophils, macrophages and T_H_2 cells (step 4) and recognition of cancer cells by T cells (step 6) in the iCCA samples (Fig. 4a and Supplementary Fig. 8). These findings were supported by TIP analysis at the protein level (Fig. 4b). Further, specific correlations of *MARCO* with genes linked to T_H_2 response ratified the association between IL4/IL-13 producing T cells and *MARCO* expression (Fig. 4c and Supplementary Fig. 6).

### MARCO^+^ TAMs in human iCCAs are primarily located in desmoplastic areas

**Fig. 5:**
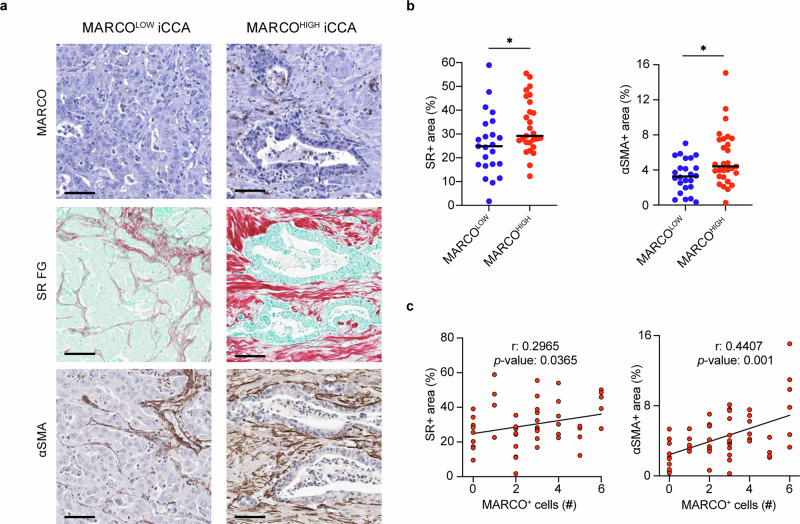
MARCO^+^ TAMs are associated with collagen deposition. **a** Representative immunohistochemical images of MARCO expression in iCCA tumors, classified as MARCO^LOW^ or MARCO^HIGH^ based on the median number of MARCO^+^ cells. Additionally, Sirius Red staining and immunohistochemical staining for α-SMA are shown in both MARCO^LOW^ and MARCO^HIGH^ iCCAs from the European CCA Histology Registry (*n* = 55). Scale bars = 75 µm. **b** Quantification of Sirius Red (SR)^+^ areas and α-SMA^+^ areas in MARCO^LOW^ and MARCO^HIGH^ iCCAs. Data are shown as median. * represents a *P* value of <0.05. **c** Correlation between SR^+^ areas and the number of MARCO^+^ cells, as well as the correlation between α-SMA^+^ areas and MARCO^+^ cells in iCCA tumors. Spearman’s rank correlation coefficient was used; correlation coefficient (r) and *P* value are shown. α-SMA α-smooth muscle actin, MARCO macrophage receptor with collagenous structure, SR Sirius Red

iCCA is characterized by desmoplastic stroma linked to accumulation of ECM.^37^ Noting that *MARCO*^+^ TAMs exhibited enriched gene sets related to ECM remodeling when compared to *MARCO*^+^ macrophages in non-tumor sites (healthy or SN liver) or *MARCO*^-^ TAMs (Fig. 2f, g and Supplementary Fig. 4e), we next examined the relationship between MARCO^+^ TAMs and desmoplasia in iCCA histological sections from the ENS-CCA Histology Registry. In agreement, MARCO^HIGH^ iCCA tumors were characterized by both higher collagen content and higher number of α-smooth muscle actin^+^ (αSMA^+^) cells compared to MARCO^LOW^ tumors (Fig. 5a, b). Further, the number of MARCO^+^ cells within the tumor positively correlated with Sirius Red^+^ (SR^+^) and αSMA^+^ areas (Fig. 5c). Together, these data indicate that MARCO*-*expressing TAMs are found in histopathological fibrotic lesions in iCCA tumors, which are linked to tumor progression and invasiveness.^37^

### *Marco*^*−/−*^ mice are partially protected from biliary tumorigenesis and present a reduction of cells associated with a T_H_2 response

*Marco* expression was analyzed in different primary cell types isolated from normal mouse livers. As observed in humans, *Marco* expression was mainly found in KCs (Supplementary Fig. 9).

**Fig. 6:**
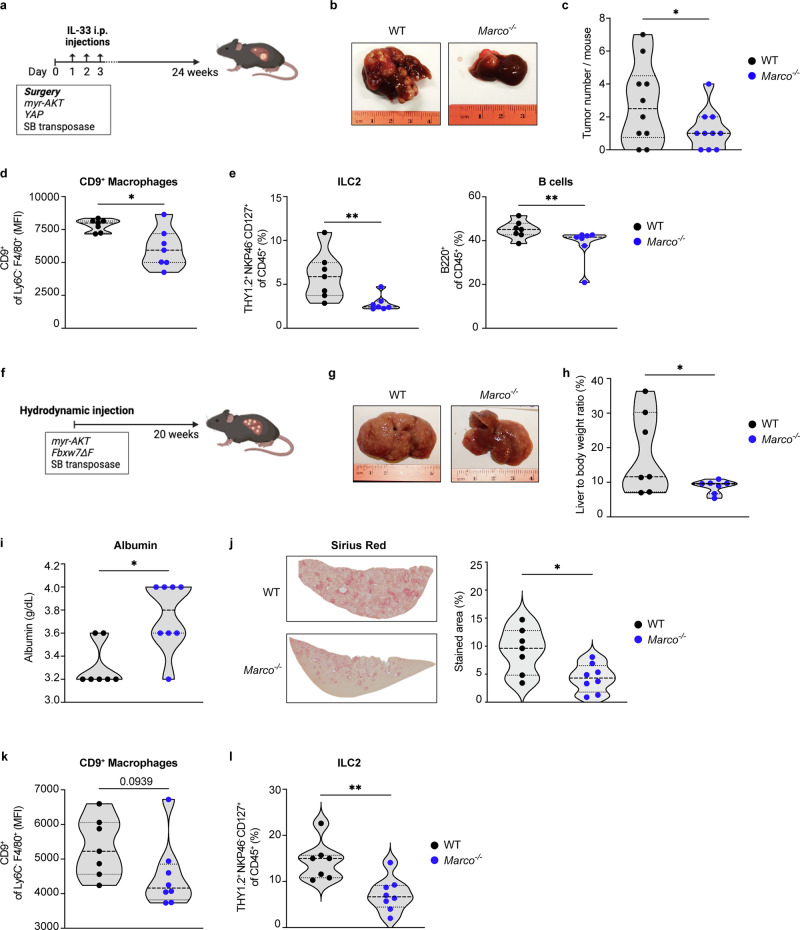
*Marco*^*−/−*^ mice are partially protected from biliary tumorigenesis. **a** WT (*n* = 10) and *Marco*^−/−^ (*n* = 11) mice were subjected to a biliary tract oncogene transduction of constitutively activated *AKT* (*myr-AKT*) and *YAP* combined with a partial bile duct ligature and subsequent systemic IL-33 administration. Mice were sacrificed 24 weeks after surgery. **b** Representative images of iCCA tumors. **c** Number of tumors per liver. **d** MFI of CD9 in liver macrophages (CD45^+^B220^-^CD3^-^LY6G^-^F480^+^LY6C^-^CD9^+^). **e** Percentage of ILC2 population (CD45^+^THY1.2^+^CD127^+^NKP46^-^) and B cells (B220^+^ CD45^+^) among total immune cells (CD45^+^). **f** WT (*n* = 7) and *Marco*^−/−^ (*n* = 8) mice received constitutively activated *AKT* (*myr-AKT*), *Fbxw7ΔF* and SB using hydrodynamic tail vein injection and were sacrificed 20 weeks later. **g** Representative images of iCCA tumors. **h** Liver-to-body weight ratios (%). **i** Serum levels of albumin (g/dL). **j** Representative images of livers stained with Sirius Red and quantification of the percentage of the stained area. **k** MFI of CD9 in liver macrophages (CD45^+^B220^-^CD3^-^LY6G^-^F480^+^LY6C^-^CD9^+^). **l** Percentage of ILC2 population (CD45^+^THY1.2^+^CD127^+^NKP46^-^) among total immune cells (CD45^+^). Data are shown as mean ± SEM. Parametric two-tailed Student’s *t* test and non-parametric Mann–Whitney test were used, except in (**c**, **h**), in which one-tailed Student’s *t* test was employed. * and ** denote *P* values of <0.05 and <0.01, respectively. ILC innate lymphoid cell, Marco macrophage receptor with collagenous structure, MFI mean fluorescence intensity

To investigate how MARCO impacts cholangiocarcinogenesis and whether it does so *via* immunosuppressive mechanisms, we next performed an oncogene-driven iCCA mouse model^38^ in WT and *Marco-*deficient (*Marco*^*−/−*^) mice (Fig. 6a). Versus WT controls, *Marco*^*−/−*^ mice showed fewer liver tumors (Fig. 6b, c) with CK19 expression confirming these tumors were biliary tumor cells (Supplementary Fig. 10a). To understand the mechanisms behind this reduced tumorigenesis, we next analyzed the hepatic immune cell landscape. Compared to WT controls, *Marco*^*−/−*^ mice exhibited lower CD9^+^ levels on their macrophages, suggesting MARCO impacts scar-associated macrophages (SAMs)^39^ (Fig. 6d and Supplementary Fig. 11a). Furthermore, there was a reduction of type 2 innate lymphoid cells (ILC2s) which are well described to produce IL-4 and IL13,^40^ as well as B cells (Fig. 6e and Supplementary Figs. 12 and 13a, b). Although *Marco*^*−/−*^ mice presented a trend toward increased NK cells, these differences were not statistically significant (Supplementary Fig. 10b). In addition, qRT-PCR revealed lower levels of *Tnf-α*, which promotes CCA invasiveness,^41^ and decreased *Il-10*, in *Marco*^*−/−*^ mice compared to WT mice (Supplementary Fig. 10c).

In a second iCCA model^42^ (Fig. 6f), although the macroscopic tumor burden was not measured due to multiple small tumors, *Marco*^*−/−*^ mice showed a lower liver-to-body weight ratio compared to WT mice (Fig. 6g, h and Supplementary Fig. 14a). *Marco*^*−/−*^ mice also had higher serum albumin levels (Fig. 6i), approaching levels seen in healthy WT mice (~4 g/dL, data not shown), suggesting better-preserved liver function under tumor-bearing conditions. Additionally, *Marco*^*−/−*^ mice exhibited a strong albeit statistically insignificant tendency towards lower hepatic transcript levels of cytokines involved in cholangiocarcinogenesis (*Il-6, Il-10*) and liver fibrosis (*Col1a1, α-SMA*) (Supplementary Fig. 14b, c). Importantly, Sirius red staining indicated that iCCA suffering *Marco*^*−/−*^ mice had lower ECM deposition, indicative of attenuated desmoplasia or fibrosis (Fig. 6j). Further, there was a similar immune profile to the previous model, as a tendency towards reduced levels of CD9 in macrophages and a reduction in the number of ILC2 cells were observed (Fig. 6k, l). *Marco*^*−/−*^ mice also had more monocyte-derived DCs and the population of DCs presented lower levels of PD-L1, suggesting enhanced antigen presentation activity (Supplementary Fig. 15a, c). Moreover, *Marco*^*−/−*^ mice had less neutrophils compared to WT counterparts (Supplementary Fig. 15b).

### *Marco*^*−/−*^ mice are protected from iCCA progression, leading to increased survival

**Fig. 7:**
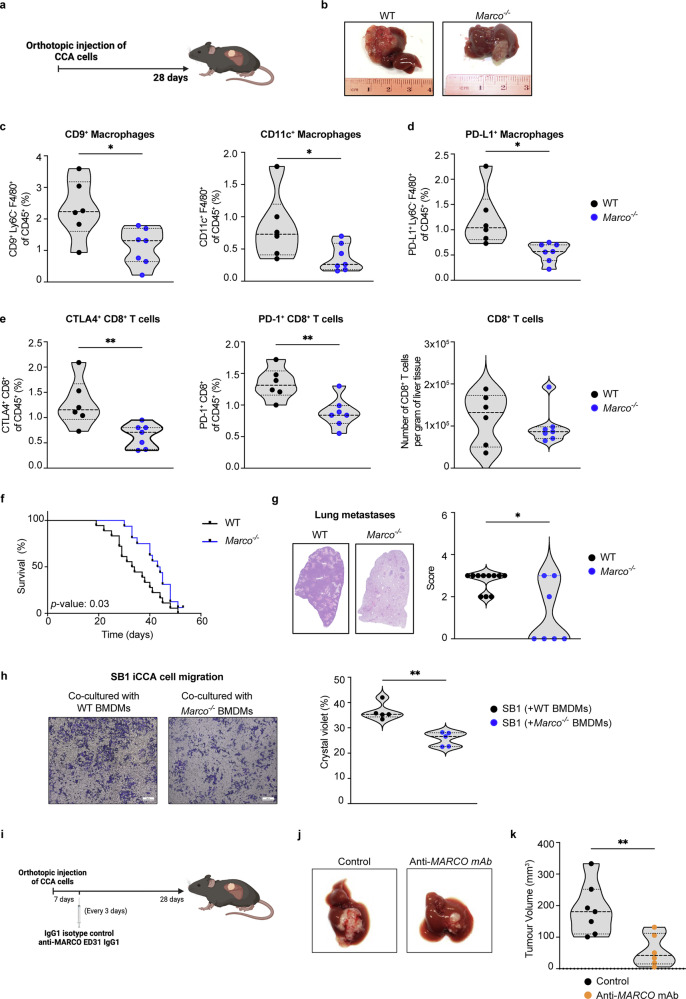
*Marco* deficiency protects mice from iCCA progression. **a** WT (*n* = 6) and *Marco*^−/−^ (*n* = 7) mice were subjected to an orthotopic CCA cell injection of 500,000 mouse SB1 CCA cells in the liver, and mice were sacrificed 28 days later. **b** Representative images of iCCA tumors in livers from WT and *Marco*^−/−^ mice. **c** Percentage of CD9^+^ macrophages (CD45^+^B220^-^CD3^-^LY6G^-^F480^+^LY6C^-^CD9^+^) and CD11c^+^ macrophages (CD45^+^B220^-^CD3^-^F480^+^CD11c^+^) among total immune cells (CD45^+^). **d** Percentage of PD-L1^+^ macrophages (CD45^+^B220^-^CD3^-^F480^+^LY6C^-^PD-L1^+^) and **e** CTLA-4^+^ and PD-1^+^ CD8^+^ cytotoxic T cells (CD45^+^CD3^+^CD8^+^) among total immune cells (CD45^+^) and number of CD8^+^ cytotoxic T cells per gram of liver tissue. **f** Kaplan–Meier curves comparing the survival of WT (*n* = 18) and *Marco*^−/−^ (*n* = 16) mice upon orthotopic injection of SB1 cells. **g** Representative images and lung metastasis scores in WT (*n* = 11) and *Marco*^−/−^ (*n* = 7) mice 21 days after surgery. **h** Representative images and quantification of crystal violet-stained areas from the SB1 iCCA cell migration assay, in which SB1 cells were co-cultured with BMDMs from WT (*n* = 5) or *Marco*^*−/−*^ (*n* = 5) mice. **i** WT mice were subjected to an orthotopic CCA cell injection of 500,000 mouse SB1 CCA cells in the liver. Seven days later, mice were treated with a rat IgG1 isotype control (*n* = 7) or a rat anti-mouse MARCO antibody (ED31 IgG1) (*n* = 6) every 3 days. Mice were sacrificed 28 days later. **j** Representative images of iCCA tumors. **k** Tumor volume in the liver (mm^3^). Data are shown as mean ± SEM. Scale bar = 200 μm. Parametric Student’s *t* test and non-parametric Mann–Whitney test were used. * and ** denote *P* values of <0.05 and <0.01, respectively. CTLA-4 cytotoxic T-lymphocyte antigen 4, Marco macrophage receptor with collagenous structure, MFI mean fluorescence intensity, PD-1 programmed cell death protein 1, PD-L1 programmed cell death protein ligand 1

To further evaluate the role of MARCO in iCCA progression, a syngeneic orthotopic mouse model of iCCA was performed in both genotypes of animals (Fig. 7a, b). *Marco*^*−/−*^ mice presented a lower number of CD9^+^ scar-associated macrophages as well as a reduction of the CD11c^+^ macrophage population (Fig. 7c and Supplementary Figs. 11 and 16), which were recently described to drive hepatocyte death-triggered liver fibrosis in a murine model of metabolic-dysfunction associated steatohepatitis (MASH).^43^ Moreover, in comparison to controls, *Marco*^*−/−*^ animals had fewer PD-L1^+^ macrophages (Fig. 7d and Supplementary Fig. 17). Although we noted no differences in the total number of CD8^+^ T cells between the genotypes, *Marco*^*−/−*^ animals had a smaller percentage of CTLA-4^+^ and PD-1^+^ CD8^+^ T cells (Fig. 7e and Supplementary Fig. 13). These data suggested a reduced immunosuppressive environment in *Marco*^*−/−*^ mice that could lead to attenuated tumor progression.

Confirmatory, in this experimental iCCA model, compared to controls, *Marco*^*−/−*^ mice exhibited improved OS (Fig. 7f), which was associated with fewer lung metastases (Fig. 7g). Noting the heterogeneus nature of MARCO^+^ TAMs (Fig. 2e and Supplementary Fig. 3a), we next sought to examine how they might affect iCCA behaviour by co-culturing SB1 cells with *Marco*^*−/−*^ versus WT KCs or bone marrow-derived macrophages (BMDMs). Interestingly, the aforementioned differences in metastatic burden observed in *Marco*^*−/−*^ mice could, at least in part, be explained by changes identified in the proteomic profile of SB1 iCCA cells co-cultured with *Marco*^*−/−*^ cells (Supplementary Fig. 18a, e). Relative to SB1 iCCA cells co-cultured with WT KCs or BMDMs, those cultured with MARCO deficient cells showed alterations in pathways related to cytoskeletal organization and cell migration (Supplementary Fig. 18b–d,f–h). Confiriming MARCO deficiency negatively impacted iCCA cell motility, SB1 cells co-cultured with *Marco*^*−/−*^ BMDMs displayed a reduced migratory capacity versus those co-cultured with WT controls (Fig. 7h).

Importantly, WT mice subjected to this orthotopic model and treated with the anti-MARCO mAb ED31^15,44,45^ showed reduced tumor volume compared to mice receiving an isotype control (Fig. 7i–k).

## Discussion

Immunotherapy has recently emerged as a promising strategy to treat cancer.^46^ In CCA, combination therapies with durvalumab, an anti-PD-L1 monoclonal antibody, or pembrolizumab, an anti-PD-1 monoclonal antibody, in combination with gemcitabine and cisplatin are the preferred first-line treatment options for advanced stages.^5,6^ Noteworthy, patients with microsatellite instability (MSI), mismatch repair deficiency (dMMR) and/or high tumor mutational burden (hTMB) have shown the most promising responses to immune checkpoint inhibitors.^47,48^ However, the proportion of CCA patients with these characteristics is low. Additionally, only a small proportion of CCAs display features of immune activation, as evidenced by lymphocyte infiltration and the expression of immune checkpoint molecules. A recent classification of iCCAs indicates that ~65% of tumors resemble “cold tumors”, characterized by a lower presence of CD8^+^ T cells and a predominance of immunosuppressive cells, such as M2-type TAMs and regulatory T cells (Tregs).^8^ TAMs are considered as the most abundant immune cells in the TME and targeting TAMs in solid tumors is emerging as a promising therapeutic strategy.^49^ However, to date no specific targets for reprogramming TAMs in iCCA have been identified.^50^ In this study, single-cell transcriptomic profiling revealed that *MARCO* is predominantly expressed in macrophages in healthy and cirrhotic livers, as well as in TAMs of iCCA tumors. In line, the presence of MARCO^high^ macrophages in iCCA tumors has recently been reported.^51,52^ Our data are also in agreement with previously published work in other solid tumors such as breast cancer and melanoma.^15^

Analysis of *MARCO* gene and protein expression in large cohorts of human iCCA demonstrated *MARCO* expression is lower in tumor tissues compared to adjacent non-tumoral liver tissues. However, some patients presented intratumoral infiltration of *MARCO*^*+*^ macrophages, likely due to a higher infiltration of innate immune cells into the tumor. Building on these observations, we explored the mechanisms underlying MARCO induction in TAMs. Our data suggest that MARCO expression is driven by T_H_2 cytokines within the TME. In vitro, IL-4 and IL-13 lead to *MARCO* upregulation within macrophages. Single-cell RNA sequencing of human iCCA tumors identified T cells as robust sources of IL-4 and IL-13, while malignant cholangiocytes, macrophages, and CAFs showed no detectable expression. Nonetheless, although iCCA cells do not produce these cytokines directly, they are established to promote a T_H_2-skewed microenvironment,^53^ which in turn would lead to MARCO expression in TAMs.

Consistent with previous reports identifying MARCO as a dismal prognostic marker in other cancers such as glioblastoma, pancreatic cancer and breast cancer,^11–13^ our findings demonstrate elevated *MARCO* expression levels correlated with worse OS in several independent cohorts of patients with iCCA following surgical resection. These data align with very recent findings demonstrating that MARCO^+^ TAMs are associated with poor iCCA prognosis, underscoring the value of MARCO as a prognostic biomarker in iCCA.^52^

When characterizing *MARCO*^*+*^ TAMs in iCCA, and in agreement with studies in hepatocellular carcinoma,^54^ we found that they express both KC and monocyte-derived macrophage markers, suggesting a heterogeneous origin. Given this mixed origin and the absence of TAM-specific Cre mouse lines, we opted to use MARCO full-body KO mice to comprehensively assess MARCO function in both KCs and monocyte-derived macrophages that contribute to the TAM pool. Notably, when comparing *MARCO*^+^ TAMs to *MARCO*^+^ macrophages in non-tumor sites (surrounding and healthy liver), our results indicate that *MARCO*^+^ TAMs acquire proliferative and ECM remodeling capacity^55^ while losing homeostatic and immunological functions typically attributed to KCs, including complement cascade activity, heme scavenging and binding/uptake of ligands by scavenger receptors.^30–32^ Reactome pathway analysis of dysregulated genes between *MARCO*^*+*^ and *MARCO*^*-*^ TAMs indicated that upregulated genes in *MARCO*^*+*^ TAMs were linked to neutrophil degranulation, a process associated with immunosuppression in other solid carcinomas.^28^ Notably, galectin-3 (*LGALS3*), reported to be secreted by macrophages to stimulate neutrophil degranulation, was among these genes.^56^ *MARCO*^+^ TAMs were also associated with IL-10 signaling. Accordingly, a recent study examining the spatial heterogeneity of liver resident macrophages revealed that *Marco* was exclusively expressed in a subpopulation of IL-10-producing macrophages located in periportal vein (PV) zones with immunosuppressive properties.^16^ Besides, reactome analysis revealed that downregulated genes in *MARCO*^*+*^ TAMs were involved in IFN-γ signaling, which is known to boost anti-tumor responses.^57^

In the same line, the TIDE algorithm further indicated that MARCO^HIGH^ iCCA tumors are associated with T cell dysfunction, characterized by a reduced proliferative capacity, decreased effector function and overexpression of multiple inhibitory receptors.^58^ Of note, MARCO positively correlated with inhibitory receptors expressed in dysfunctional T cells, including PD-1, CTLA-4, BTLA, HAVCR2 and TIGIT. Further, spatial proteomics analysis identified a profound upregulation of almost all tested immunosuppressive markers in both MARCO^+^ and MARCO^-^ regions of interest (ROIs). Notably, versus MARCO^−^ areas, MARCO^+^ ROIs exhibited elevated PD-L1 protein expression, known as a immunosuppressive marker for T cells. These data, together with data demonstrating less PD-L1 in MARCO-deficient macrophages of iCCA suffering animals along with reduced PD-1 and CTLA-4 expression in cytotoxic CD8^+^ T cells strongly indicate that within the TME, MARCO contributes to the upregulation of immunosuppressive molecules and T cell dysfunction. Recent work supports these findings, demonstrating a reduced presence of CD3^+^ and CD8^+^ T cells, accompanied by higher expression of immune checkpoints such as PDCD1, CTLA-4, and TIGIT in MARCO^HIGH^ regions.^52^

We observed that MARCO^+^ TAMs were found in histopathological fibrotic lesions in human iCCA samples, suggesting MARCO may be linked to fibrosis as well as tumor progression and invasiveness through ECM remodeling. Employing different experimental models of iCCA in WT and *Marco*^−/−^ mice^38,42^ revealed that compared to WT controls, *Marco*^−/−^ mice showed partial protection from tumorigenesis as well as a notable reduction of CD9^+^ expression in macrophages. Interestingly, CD9^+^ scar-associated macrophages are linked to fibrosis development.^39^ Demonstrating the relevance of these findings to human iCCA, scRNA-seq analysis revealed that *MARCO*^+^ TAMs were enriched in a variety of scar-associated macrophage markers,^59^ including *TREM2*, *LGALS3*, *IL1B and SPP1*. Further, *MARCO*^*+*^ TAMs were enriched for genes associated with the assembly of collagen fibrils and ECM remodeling, including cathepsins^60^ and versican (*VCAN*),^61^ suggesting MARCO might play a role in liver fibrosis and/or tumor desmoplasia.

We also found a decrease in B cells and ILC2s in *Marco*^−/−^ mice. Despite representing a small group of cells in the healthy liver, ILC2s can expand in the disease setting and activate a T_H_2 immune response by secreting different cytokines, including IL-4 and IL-13, therefore modulating the TME.^62^ In this regard, it has also been described that B cells drive T_H_2 responses by instructing human dendritic cell maturation.^63^ Thus, these findings support the idea that in the absence of MARCO, there might be a reduced T_H_2 response that results in diminished cholangiocarcinogenesis. Of note, there were no differences in the tested immune cell populations at steady state between genotypes (data not shown).

In patients with advanced tumor stages (stage IV) and therefore metastasis, higher MARCO expression was observed, suggesting a potential role of MARCO in tumor progression. In fact, in an orthotopic CCA model *Marco*^−/−^ mice presented less metastatic *foci* in the lungs and a higher median survival (43.5 days) than WT mice (33.5 days). Importantly, *Marco*^−/−^ mice presented a lower number of macrophages and cytotoxic T cells that express immune checkpoint molecules, as well as a lower percentage of CD9^+^ macrophages compared to WT mice, supporting the notion that loss of MARCO promotes a multicellular, pleiotropic anti-tumor response, involving less exhausted PD-1 and CTLA-4^+^ CD8^+^ T cells, reduced immunosuppressive signaling, and improved immune access of cell types and secreted cytokines to the tumor microenvironment. Interestingly, SB1 iCCA cells co-cultured with *Marco*^*−/−*^ macrophages demonstrated reduced migratory capacity, highlighting that MARCO not only influences tumor growth but also prevents dissemination. Of note, treatment with anti-MARCO antibody reduced the tumor volume in a syngeneic orthotopic mouse model of iCCA. This finding is aligned with other studies showing that anti-MARCO monoclonal antibodies can reprogram TAMs into a pro-inflammatory phenotype, thereby enhancing tumor immunogenicity in various cancers, including hepatocellular carcinoma.^14,15^ Our results suggest that targeting MARCO might be considered a promising new targeted immunotherapy for iCCA patients, alone or in combination with chemotherapeutic and/or immunotherapeutic regimens, which warrants further investigation.

**Fig. 8:**
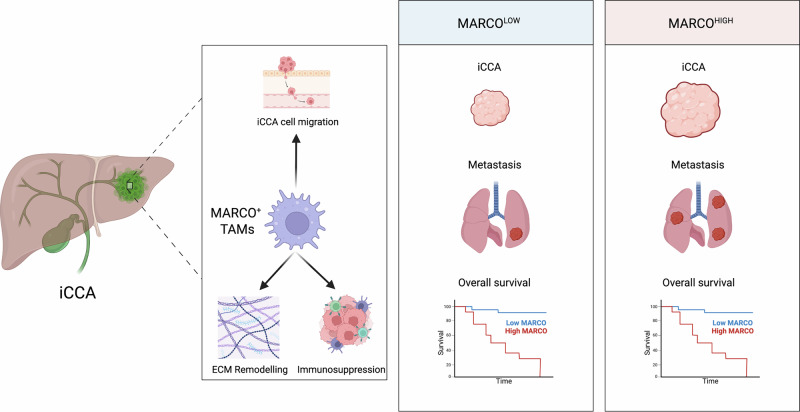
Graphical abstract. MARCO is expressed in TAMs within the iCCA tumor microenvironment. MARCO⁺ TAMs contribute directly, by promoting iCCA cell motility and migration, and indirectly, by remodeling the ECM and suppressing anti-tumor immunity, thereby fostering a protumorigenic niche. Notably, compared to MARCO^LOW^ iCCA tumors, MARCO^HIGH^ iCCAs are associated with increased tumor burden, enhanced metastatic potential, and significantly reduced overall survival. Created with BioRender.com

In summary, findings consistent across both human data and experimental mouse models indicate that MARCO is expressed in TAMs within the iCCA tumor microenvironment and plays a central role in driving tumor progression. MARCO⁺ TAMs contribute directly, by promoting cancer cell motility and migration, and indirectly, by remodeling the ECM, shaping a Th2-skewed and immunosuppressive microenvironment, and modulating immune checkpoint expression, thereby establishing a protumorigenic niche. This immunosuppressive environment is particularly evident in MARCO^HIGH^ iCCA tumors, which are associated with increased tumor burden, enhanced metastatic potential, and significantly reduced overall survival compared to MARCO^LOW^ iCCA tumors (Fig. 8). Nevertheless, we acknowledge that fully dissecting the molecular mechanisms underlying MARCO⁺ TAM function remains a limitation of our study. While our integrated approach provides robust evidence for MARCO’s role in promoting cancer cell motility, ECM remodeling and immunosuppression, future studies employing co-culture of flow cytometry sorted MARCO^+^ TAMs with T cells, fibroblasts and cholangiocytes will be required to uncover precise signaling pathways. Altogether, our results suggests that MARCO is a promising target for the development of new therapeutic strategies to overcome the immunosuppressive barriers in iCCA.

## Methods

### Resource availability

Further information and requests for resources and reagents should be directed to and will be fulfilled by the corresponding author, María J. Perugorria (majesus.perugorriamontiel@bio-gipuzkoa.eus).

### Experimental model and study participant details

#### Patient samples

Human iCCA tumors, surrounding non-tumor liver and/or normal bile ducts from six independent cohorts of patients [Copenhagen (Denmark; GSE26566), Job (France; E-MTAB-6389), Jusakul (Asia, Europe, South America; GSE89749), Nakamura (Japan; EGA00001000950), Dong (China; OEP001105) and The Thailand Initiative in Genomics and Expression Research (TIGER) (Thailand; GSE76311)] were studied at the transcriptomic level. For histomorphological and immunohistochemical analyses, samples from the European CCA Histology Registry, endorsed by the European Network for the Study of Cholangiocarcinoma (ENS-CCA) were employed. The use of human biological samples was approved by the Ethics Committee of Sapienza University of Rome (Code: 4492).

#### Single-cell RNA sequencing

Publicly available single-cell RNA sequencing data from healthy livers and cirrhotic livers (GSE136103) and from CCA tumor and surrounding tissue samples (GSE125449; GSE138709; GSE151530) were employed. Cell markers used for the annotation of clusters are included in the supplementary information.

#### Experimental models of CCA

Oncogene-driven iCCAs and an orthotopic experimental model were generated and employed as described in the supplementary information. Experiments were performed in WT and *Marco*^*−/−*^ mice on a C57BL/6 genetic background. Mouse experimental models of CCA were performed under the approval of the Animal Experimentation Ethics Committee of Biogipuzkoa Health Research Institute and Diputación Foral de Gipuzkoa (CEEA20/09 OH20-23 PRO-AE-SS-189; CEEA20/17 OH21-03 PRO-AE-SS-197; CEEA21/17 OH21-41 PRO-AE-SS-236; CEEA23/09 OH23-22 PRO-AE-SS-307). Our study exclusively examined male mice.

#### Mouse primary cell isolation

Hepatocytes, cholangiocytes, KCs and HSCs were isolated as previously reported.^64,65^ Liver mononuclear cell isolation and preparation are described in the supplementary information.

#### Histological analyses

H&E staining was performed to analyze tissue morphology in human and mouse samples. Detection of MARCO protein expression by IHC was carried out on paraffin-embedded sections from normal surrounding and CCA human liver tissue as described in the supplementary information.

#### RNA isolation and gene expression

Total RNA was isolated from both cells in culture and mouse liver samples using TRI Reagent®, following the manufacturer’s instructions. Subsequently, reverse transcription and quantitative real-time PCR were conducted as described in the supplementary information.

#### Mass spectrometry and proteomic analysis

Comparative shotgun proteomic analyses of SB1 cells, and WT and *Marco*^*−/−*^ KCs and BMDMs were performed as described in the supplementary information.

#### Statistical analysis

Statistical analyses were performed as described in the supplementary information.

## Supplementary information

Supplemental material

## Data Availability

The proteomic datasets generated during this study have been deposited in the MassIVE repository under the accession number MSV000100316.
