# Supplementary material for: MARCO promotes cholangiocarcinogenesis by inducing immunosuppression and its targeting reduces tumor growth
Source: Signal Transduct Target Ther. 2026 Apr 29;11:158. doi: 10.1038/s41392-026-02657-w (PMC13125608; doi:10.1038/s41392-026-02657-w)
Supplement: Supplementary file 1 — Supplemental material [file 41392_2026_2657_MOESM1_ESM.docx]

Supplementary Materials for

**MARCO promotes cholangiocarcinogenesis by inducing immunosuppression and its targeting reduces tumor growth**

Aloña Agirre-Lizaso,^1^ Maider Huici-Izagirre,^1^ Colm J. O’Rourke,^2^ Ekaterina Zhuravleva,^2^ Guido Carpino,^3^ Diletta Overi,^3^ Josu Urretabizkaia-Garmendia,^1^ Ibone Labiano,^1,4^ Ana Korosec,^5,6^ Beatriz Val,^1,7^ Diego F. Calvisi,^8^ Sumera I. Ilyas,^9^ Joke M.M. den Haan,^10^ Patricia Aspichueta,^7,11,12^ Elizabeth Hijona^1,7^, Adelaida La Casta,^1^ Raúl Jiménez-Agüero,^1^ Ana Lleo,^13,14^ Rocio I.R. Macias,^7,15^ Eugenio Gaudio,^3^ European CCA Histology Registry,^§^ Jesper B. Andersen,^2^ Gernot Schabbauer,^5,16^ Luis Bujanda,^1,7^ Pedro M. Rodrigues,^1,7,17^ Omar Sharif,^5,6^ Jesus M. Banales,^1,7,17,18,*, ‡^ Maria J. Perugorria.^1,7,19,*, ‡^

Correspondence to: majesus.perugorriamontiel@bio-gipuzkoa.eus (M.J.P.), jesusmaria.banalesasurmendi@bio-gipuzkoa.eus (J.M.B.).

**This PDF file includes:**

Materials and Methods

Figures. S1 to S18

Tables S1 to S5

References

European CCA Histology Registry

Materials and Methods

**Human samples**

Human iCCA tumors, surrounding non-tumor liver and/or normal bile ducts from six independent cohorts of patients [Copenhagen (Denmark), Nakamura (Japan), Job (France), Jusakul (Asia, Europe, South America), Dong (China), San Sebastian (Spain) and The Thailand Initiative in Genomics and Expression Research (TIGER) (Thailand)] were studied.

Copenhagen cohort

Whole transcriptome profiling [humanRef-8v2 BeadChips (Illumina Inc)] was performed in the samples of the Copenhagen cohort, which included a total of 151 iCCA surgical specimens, 143 normal surrounding liver samples, and 9 normal intrahepatic bile ducts (GEO: GSE26566).^1,2^

Nakamura cohort

A combination of whole-exome and transcriptome sequencing was performed in a large cohort of patients, including 145 iCCA samples.^3^

Job cohort

78 iCCA samples and their respective 31 distant non-tumor tissues were subjected to bulk-tumor RNA gene expression profiling. The transcriptome profiling was performed with the Affymetrix Human Transcriptome Array 2.0 (ArrayExpress, E-MTAB-6389).^4^

Jusakul cohort

Transcriptome and survival data from 81 iCCA samples were obtained (GSE89749).^5^ Patient samples were obtained from Singapore, Romania, Thailand, Italy, France, South Korea, Brazil, Taiwan, China and Japan.

Dong cohort

Transcriptome and survival data from 244 iCCA samples were obtained (OEP001105).^6^ Patient samples were obtained from China.

The Thailand Initiative in Genomics and Expression Research (TIGER) cohort

Affymetrix Human Transcriptome Array 2.0 was employed to perform genome-wide transcriptome profiling of 91 iCCA surgical samples and matched paired surrounding liver tissues (GSE76311).^7^

**European CCA Histology Registry**

The European CCA Histology Registry, endorsed by the European Network for the Study of Cholangiocarcinoma (ENSCCA), represents a digitalized repository of stained slides obtained from individuals who underwent liver resection with curative intent for CCA. Samples are matched with clinical and serological information obtained from the INT-CCA registry. For this study, n=55 iCCA cases were used (**Supplementary Table 1**). All research was conducted in accordance with both the Declarations of Helsinki and Istanbul. The European CCA Histology Registry protocol was approved by the Ethic Committee of Sapienza University of Rome, Rome, Italy (Code: 4492), serving as coordinating center. Additionally, each participating center secured local ethical approval (or equivalent) and finalized a Material Transfer Agreement with the Sapienza University of Rome. Written informed consent was given by all subjects. Samples and clinical data were pseudonymized.

**Single cell RNA sequencing**

Cell-type specific *MARCO* expression was examined in iCCA human tumors by utilizing publicly available single-cell RNA sequencing data from healthy livers and cirrhotic livers (GSE136103),^8^ and from iCCA tumor and surrounding tissue samples (GSE125449; GSE138709; GSE151530). Analysis was conducted with Seurat R package. Individual processing of the datasets was performed as follows:

GSE136103 dataset

Data were processed with Seurat version 3.2.3.; Filtering for high quality cells was carried out selecting features reported at least in 3 cells and cells with at least 300 features. Cells with UMI counts below 1000 or mitochondrial content above 20% were removed. Data was normalized with LogNormalize transformation and scale factor 10000, and scaled according to default Seurat settings. In GSE136103, normal and cirrhotic livers were processed separately with 2000 top variable features considered for principal component analysis (PCA) and top 15 PCA components were selected to perform dimension reduction.

GSE125449 dataset

Data were processed with Seurat version 4.0.0.; Filtering was carried out selecting features reported at least in 3 cells and cells with at least 500 features. Cells with UMI counts below 700 or mitochondrial content above 20% were removed. Outliers were defined per sample with scatter and removed. Doublets/multiplets were predicted and removed with scDblFinder using default settings. Data was scaled with LogNormalize transformation and scale factor 10000 according to default Seurat settings. Variable genes for principal component analysis were identified using the Seurat function FindVariableFeatures and applying the vst method with 2000 features selected. Data was scaled, and UMI number and mitochondrial gene content were regressed out. Top 30 PCA components were selected to perform dimension reduction.

Regarding the TAMs analysis, Harmony R package was used for data integration with donor considered as batch. Top 6 PCA components were selected to perform dimension reduction. Logistic regression framework was used to identify differentially expressed genes with consideration of donor as a latent variable. Genes with highest differences between groups were defined as having average log2FC >1.5 and adjusted *p*-value <0.01 and were included in the heatmap.

GSE138709 dataset

Data were processed with Seurat version 4.0.0.; Features reported at least in 3 cells and cells with at least 500 features were considered. Cells with UMI counts below 700 or mitochondrial content above 35% were removed. Outliers were defined per sample with scatter and removed. Doublets/multiplets were predicted and removed with scDblFinder using default settings. Data were scaled with LogNormalize transformation and scale factor 10000 according to default Seurat settings. Variable genes for principal component analysis were identified using the Seurat function FindVariableFeatures and applying the vst method with 2000 features selected. Data were scaled, and UMI number and mitochondrial gene content were regressed out. Top 30 PCA components were selected to perform dimension reduction.

For separate analysis of TAMs, Harmony R package was used for data integration with donor considered as batch. Top 16 PCA components were selected to perform dimension reduction. Logistic regression framework was used to identify differentially expressed gene with consideration of donor as a latent variable. Genes with highest differences between groups were defined as having average log2FC >1.5 and adjusted *p*-value <0.01 and were included in the heatmap.

GSE151530 dataset

Data were processed with Seurat version 4.0.0.; Features reported at least in 3 cells and cells with at least 500 features were considered. Cells with UMI counts below 700 or mitochondrial content above 35% were removed. Outliers were defined per sample with scatter and removed. Doublets/multiplets were predicted and removed with scDblFinder, default settings. Data were scaled with LogNormalize transformation and scale factor 10000 according to default Seurat settings. Variable genes for principal component analysis were identified using the Seurat function FindVariableFeatures and applying the vst method with 2000 features selected. Data were scaled, and UMI number and mitochondrial gene content were regressed out. Top 30 PCA components were selected to perform dimension reduction.

These datasets were annotated encompassing both malignant and non-malignant cell types.^9–11^ Cell markers used for the annotation of clusters included; T cells: CD3E, CD3D, CD3G, B cells: CD79A, FCRL5 TECs: VWF, CDH5; CAFs: COL1A2, FAP, PDPN, DCN, COL3A1, COL6A1; TAMs: CD14, CD163, CD68, CSF1R; HPC like: EPCAM, KRT19, CD24; Malignant cells (carcinoma): KRT19, EPCAM, SOX9; Dendritic cells: CD1C, CLEC9A; Cholangiocytes: FXYD2; Hepatocytes: ASGR1, APOC3, FABP1, APOA1; NK cells: KLRF1, GZMB, GZMK, PRF1. Clustered data was visualized using t-distributed Stochastic Neighbour Embedding (t-SNE) through the Seurat R package.

Regarding the separate analysis of TAMs, Harmony R package was used for data integration with donor considered as batch. Top 10 PCA components were selected to perform dimension reduction. *MARCO*^+^ clusters were defined based on *MARCO* average expression and percent of cells expressing *MARCO* in the cluster (avg.exp > 0.7, pct.exp > 10). Logistic regression framework was used to identify differentially expressed gene with consideration of donor as a latent variable. Genes with highest differences between groups were defined as having average log2FC >1.5 and adjusted *p*-value <0.01 and were included in the heatmap. To explore the functional pathways associated with genes differentially expressed between *MARCO*^+^ and *MARCO*^−^ TAMs, the Reactome Pathway Database was employed (https://reactome.org/). Pathways with a *p*-value <0.01 were considered.

To compare *MARCO*^+^ TAMs in the tumor microenvironment (TME) with *MARCO*^+^ macrophages in non-tumor sites, liver focused single cell studies were merged (GSE125449, GSE151530, GSE146409, GSE138709, GSE136103, GSE149614), and macrophages populations subsetted and re-integrated with integration anchors approach from Seurat (SCT normalized assay by dataset, 3000 variable features, 35 PCA), HCC surrounding liver dataset GSE149614 was excluded, as well as GSE146409 due to low macrophages number. GSEA results were prepared for visualization with a focus on specific pathways, selected based on statistical significance, normalized enrichment scores (NES), and differences between experimental conditions [e.g., *MARCO*^+^ vs *MARCO*^–^, healthy liver (HL) vs surrounding liver (SL)]. Only pathways from the Reactome and KEGG databases with adjusted p-value < 0.01 and absolute NES > 0.2 were considered significant. From these, the top 5 upregulated and top 5 downregulated pathways were selected for each comparison group (HL, SN, and both vs Tumor). To further refine the set, only pathways showing a NES difference greater than 1 between *MARCO*^+^ and *MARCO*^–^ were retained. Additionally, any pathway with a NES difference greater than 2 between *MARCO*^+^ and *MARCO*^–^ was included, and two keratinization related pathways were excluded.

**Consensus^TME^ tool**

The relative abundance of non-cancerous cell populations in the tumor microenvironment from iCCA was evaluated and associated to *MARCO* expression using the Consensus^TME^ tool.^12^ Consensus^TME^ compiles the cell-type specific gene sets used by other six published tumor microenvironment estimation methods. For that purpose, transcriptome data from the Jusakul cohort of patients was employed.^5^

**Tumor immune dysfunction and exclusion (TIDE) tool**

Immune functionality and associated biological features were evaluated in the iCCA tissue transcriptome datasets from the Copenhagen^1,2^ and Dong cohorts^6^ using the TIDE tool.^13^ Briefly, for each transcriptome dataset, expression values were log2-transformed after addition of a pseudocount of 1 to each value. For each individual gene, mean gene expression across all samples was subtracted from the gene expression in each individual sample. TIDE calculations were performed using the TIDE webserver (http://tide.dfci.harvard.edu/) and TIDE features were associated with *MARCO* expression by Spearman correlation analysis.

**Tumor immunophenotype profiling (TIP) tool**

The functionality of each step of the cancer-immunity cycle in iCCA samples according to *MARCO* expression was predicted using the TIP tool^14^ in two different cohorts of patients.^5,6^ Processed and normalized transcriptome and proteome data were uploaded to the TIP server (http://biocc.hrbmu.edu.cn/TIP/) with TCGA-CHOL specified as the reference cancer type. TIP features were associated with each step of the cancer-immunity cycle by Spearman correlation analysis.

***In vivo* iCCA models**

The role of *Marco* was studied *in vivo* by using sleeping beauty-based genetic mouse CCA models or a syngeneic orthotopic mouse model of iCCA. All experimental procedures were approved by the *Ethical Committee for Animal Experimentation* of the supporting Institutions and were used in conformity with the Institution’s guidelines for the use of laboratory animals. [CEEA20/09, CEEA20/17, CEEA21/17 and CEEA23/09 (Biogipuzkoa, San Sebastian)]. Animal experiments were performed in age-matched male C57BL/6 *wild-type* (WT) and *Marco* knockout (*Marco*^-/-^) mice bred at Biogipuzkoa Research Institute. *Marco*^-/-^ mice backcrossed into a C57BL/6 background were obtained from our collaborators from the Medical University of Vienna.

Murine model of iCCA based on *AKT* and *YAP* overexpression in the biliary tree

The genetic murine model of iCCA was generated in WT and *Marco*^-/-^ C57BL/6 mice based on a published study.^15^ Briefly, under deep anesthesia, the bile duct draining the left lateral liver lobe was identified and a unilobar ligature with a 6.0 silk was placed loosely around the duct. Additionally, another ligature was placed loosely around the base of the gallbladder where it meets the cystic duct. Thereafter, the common bile duct was clamped to maintain the solution in the biliary tract, and ectopic oncogene expression was accomplished by the injection in the gallbladder of the *sleeping beauty* transposon transfection system (2.5 µg) with plasmids containing constitutively active *AKT* (*myr-AKT*) and *YAP* oncogenes (11.25 µg, each) in *in vivo* jetPEI (Polyplus, New York, NY) transfection reagent. Finally, the ligatures placed around the ducts were tied and a cholecystectomy performed. Starting on postoperative day 1, for 3 days, each animal was injected with 1μg of IL-33 (R&D, Minneapolis, MN). After 24 weeks, mice were sacrificed.

Murine model of iCCA based on *AKT* and *Fbxw7ΔF* overexpression in the liver

The sleeping beauty model of iCCA was generated by the administration of 40 µg *Fbxw7ΔF*, a dominant negative form of the tumor suppressor gene *Fbxw7*, and 10 µg of an activated form of *AKT* (*myr-AKT*) plasmids together with 2.5 µg of the *sleeping beauty* transposase through hydrodynamic tail vein injection (10% of the total mouse weight) in WT and *Marco*^-/-^ C57BL/6 mice.^16^ After 20 weeks, mice were sacrificed.

Syngeneic orthotopic mouse model of iCCA

In short, murine CCA cells (SB1) derived from a genetic murine model of iCCA^15^ were obtained on a collaborative basis from Dr. Sumera I. Ilyas and these were cultured as previously described.^17^ Under deep anesthesia, using a 100 µl Hamilton syringe (ref: 1710 LT, 10333901, ThermoFisher) and a 27-gauge needle, 40 µl of DMEM 10% FBS 1%P/S containing 500,000 cells were slowly injected in the superolateral aspect of the medial lobe of the liver. Afterwards, a cotton tipped applicator was held over the injection site to prevent cell leakage and blood loss. Four weeks following SB1 cell implantation, mice were sacrificed. In order to assess differences in the lung metastases, mice were sacrificed three weeks following SB1 cell implantation.

For survival studies, WT and *Marco*^-/-^ mice were subjected to the SB1 CCA cell implantation in the superolateral aspect of the medial lobe of the liver as aforementioned. Survival was monitored twice every day.

To evaluate the effect of the anti-mouse MARCO antibody *in vivo*, WT mice were subjected to the SB1 CCA cell implantation as previously described. One week after the surgery, mice were treated every three days during 3 weeks with the rat IgG1 isotype control (BioXCell) or 100 µg of the anti-mouse MARCO antibody ED31 IgG1 (kind gift from Dr. den Haan).^18^ Four weeks following SB1 cell implantation, mice were sacrificed.

**Isolation of liver cells for flow cytometry**

Liver mononuclear cells were isolated from sleeping beauty-based genetic mouse CCA models or orthotopically implanted mouse iCCAs. After mice were sacrificed, livers were removed, excised with scalpels and digested *in vitro* for 1 hour at 37ºC in RPMI (Gibco) supplemented with 0.05% collagenase and dispase (Roche) and 0.01% trypsin inhibitor (Gibco). Then, the digested liver was filtered through a 40 μm cell strainer (Falcon), centrifuged at 800g for 10 minutes at 4ºC and resuspended in 10 ml of RPMI media. The resulting cell suspension was afterwards overlayed into 15 ml of 33% Percoll (Sigma-Aldrich) solution and centrifuged at 800g during 30 minutes at RT without brake. Afterwards, in order to lyse the erythrocytes, the cell pellet was resuspended in 2 ml of red blood lysis buffer for 3 minutes. Cells were then washed and resuspended in RPMI. Total cell number per liver was counted using a hemocytometer and cell viability was assessed using Trypan Blue dye (Lonza). Finally, cells were incubated with antibodies (**Supplementary Table 2**) during 15 minutes, fixed with the IC fixation buffer (Invitrogen), resuspended in PBS 1X and analyzed by flow cytometry (CytoFLEX S flow cytometer, Beckman Coulter).

**Liver histology and staining of murine samples**

Tissue samples were collected and fixed in 4% formaldehyde for 24 hours. Subsequently, tissues were processed using the MTM tissue processor (Slee Medical GmbH), embedded in paraffin (Thermo Fisher Scientific) and cut using the HM355S microtome (Thermo Fisher Scientific) in sections at a thickness of 4-5 μm.

Haematoxylin and eosin (H&E) staining and histology scoring

With the aim of analyzing the tissue morphology, hematoxylin and eosin (H&E) staining was performed. For that purpose, human and mouse paraffin-embedded tissue sections were deparaffinized by carrying out 2 incubations of 5 minutes in xylene (VWR). Then, samples were rehydrated with solutions of decreasing alcohol concentration (100%, 96%, 70%, 50%) for 5 minutes, with a final washing step in PBS. Slides were incubated in Harris Hematoxylin (Merck) for 5 minutes, washed in tap water, incubated in Eosin (Merck) for 5 minutes and washed in tap water again. Afterwards, slides were dehydrated with ethanol solutions of increasing concentrations (50%, 70% and 100%) for 5 minutes and incubated twice in xylene for 2 minutes. Finally, slides were mounted with Pertex (Sigma-Aldrich) and analyzed under an Eclipse 80i (Nikon) microscope. Mouse liver and lung histology was also studied by an experienced pathologist from the Department of Animal Medicine and Surgery, Veterinarian faculty, Complutense University in Madrid (Spain).

Sirius Red staining

For collagen visualization, Sirius red staining was performed on FFPE slides. Slides were deparaffinized in xylene and rehydrated. Thereafter, slides were stained with 2% phosphomolybdic acid (Sigma-Aldrich) for 2 minutes and washed with dH_2_O. Collagen was stained by incubation in 0.1% Sirius red F3BA (Gibco) for 3 hours. Finally, slides were incubated with 0.01 N hydrogen chloride (HCl) (Merck) for 2 minutes, dehydrated and mounted with DPX.

Immunohistochemistry (IHC) and image analysis

Immunostaining was performed on paraffin-embedded mouse and human liver tissue sections. In brief, the 4-5 μm thick sections were deparaffinized with xylene and rehydrated with decreasing concentrations of ethanol in water. Endogenous peroxidase activity was blocked by incubating slides in 0.6% hydrogen peroxide (H_2_O_2_) in methanol for 15 minutes. Antigen retrieval was achieved by using the appropriate method with the subsequent 30 minutes of cooling at room temperature. Afterwards, tissue slides were mounted into the sequenza system and endogenous avidin and biotin was blocked using a blocking kit (Vector Laboratories) for 20 minutes each at RT. Then, the sections were blocked with the corresponding blocking serum for 30 minutes at RT, which was derived from the animal species where the secondary antibody was generated. Primary antibodies were applied at the appropriate concentrations overnight at 4ºC (**Supplementary Table 3**). The next day, after rinsing the slides in 1X PBS and incubating with the corresponding secondary biotinylated antibodies (**Supplementary Table 3**), the ABC tertiary reagent was added to amplify the signal (Vector Laboratories). IHC were developed with 3,3'-Diaminobenzidine (DAB) (Vector laboratories). The duration of DAB incubation was determined through pilot experiments. Finally, slides were counterstained with Harris Hematoxylin solution (ApplyChem), dehydrated through ethanol and xylene, and mounted with DPX (Sigma-Aldrich). Representative pictures were taken in an Eclipse 80i (Nikon) microscope using the Digital sight DS-U2 camera controller (Nikon) using NIS-Elements.

**Histology, immunohistochemistry and immunofluorescence of human samples**

For the present study, we retrieved for analysis histo-morphological and immunohistochemical stains included the Histological CCA Registry. Stains included hematoxylin and eosin (H&E), Sirius Red/Fast Green (SR/FG), and α-smooth muscle actin (αSMA) immunohistochemistry. Moreover, 3µm-thick blank slides were retrieved to perform immunohistochemistry and immunofluorescence stains.

For immunohistochemistry, the endogenous peroxidase activity was blocked by a 30-minute incubation in methanolic hydrogen peroxide (2.5%). Antigens were retrieved as indicated by the vendor and sections then incubated with primary antibodies (**Supplementary Table 3**) overnight at 4°C. Then, samples were rinsed twice with phosphate-buffered saline for 5 min, incubated for 30 minutes at room temperature with Dako REAL EnVision Detection System (code K5007, Dako, Agilent Technologies, Glostrup, Denmark), Diaminobenzidine (code: K3468, Dako, Agilent Technologies, Glostrup, Denmark) was used as substrate, and sections were counterstained with hematoxylin. For immunofluorescence, non-specific protein binding was blocked by 5% normal goat serum. Samples were incubated overnight with primary antibodies. Then, slides were washed and incubated for 1 hour with labeled isotype-specific secondary antibodies (anti-mouse AlexaFluor®-488, anti-goat AlexaFluor®-594; Invitrogen, Life Technologies, Carlsbad, CA, USA) and counterstained with 4,6-diamidino-2-phenylindole (DAPI) for visualization of cell nuclei. For all immunoreactions, adequate negative controls were also included.

Histological sections were analyzed in a coded fashion by two independent researchers using a light microscope (Leica Microsystems DM4500B; Leica Microsystems, Weltzlar, Germany) equipped with Leica K3C Videocam and LAS X Core software. All stained slides were also scanned by a digital scanner (Aperio Scanscope CS and FL System, Aperio Digital Pathology, Leica Biosystems, Milan, Italy) and processed by Aperio ImageScope software (v.12.4.6.5003). Slides already included in the Histological Registry were retrieved using Aperio eSlide Manager software (v12.4.0.5043).

The tumor area occupied by SR^+^ collagen fibers and by αSMA^+^ cells was quantified by image analysis algorithms on ImageScope, and expressed as percentage of the tumor area. The number of MARCO^+^ cells was quantified within the tumor mass and in surrounding liver tissue. Positive cells were counted, and data is expressed as number of MARCO^+^ cells per microscopic field at 20x. Co-expression of MARCO and CD68/CD206 at immunofluorescence was quantified on scanned slides by counting single and double positive cells and expressed as percentage.

**Spatial molecular profiling**

Based on MARCO positive cell number at immunohistochemistry analysis, n= 7 iCCA were selected for analysis on the NanoString GeoMx Digital Spatial Profiler (DSP). Four samples obtained from livers with normal histology were included as controls. FFPE slides were prepared according to manufacturer’s protocol. Slides were processed with the GeoMx Solid Tumor TME Morphology Kit (item #121300301, including fluorescent antibodies against Pan-Cytokeratin, CD45) and the GeoMx Immune Cell Profiling Panel and additional modules (item #121300101); samples were incubated with anti-MARCO antibody and with labeled isotype-specific secondary antibodies (anti-rabbit AlexaFluor®-660). Finally, nuclei were counterstained with nuclear stain SYTO13.

Whole slides were imaged using the GeoMx DSP and the integrated software suite used to select regions of interest (ROI) within the tumor mass. Within the tumors, immunofluorescence for MARCO was used to select ROIs. ROIs with no MARCO^+^ cells were designed as MARCO^-^ (N=10) and ROIs enriched with MARCO^+^ cells (used cut-off value ≥ median number per HPF identified at immunohistochemistry, see Figure 1) were considered as MARCO^+^ (N=14).

Then, within each ROI, Pan-Cytokeratin and CD45 were used for the segmentation of epithelial and immune cell fractions, from which DNA oligo probes were photocleaved and cell-type specific proteomic profiles were obtained. The readout of collected probes was performed according to manufacturer’s protocol by using the nCounter Sprint profiler. Digital Count Conversion (DDC) files were uploaded into the GeoMx DSP for mapping data to the spatial origin. Data were processed by GeoMx DSP Analysis Suite (v3.0.0113) for quality control, normalization and background correction, and expression analysis. In control tissues, portal tracts were selected (N=4 ROIs), and Pan-Cytokeratin^+^ interlobular bile ducts and CD45^+^ cells were segmented.

**Primary cell isolation and experimental conditions**

Cholangiocytes, hepatocytes, Kupffer cells (KCs) and hepatic stellate cells (HSCs) were isolated as previously reported^19,20^ and gene expression analyses were performed in baseline conditions.

For co-culture experiments, KCs were isolated from WT and *Marco*^-/-^ mice as aforementioned and experiments were performed 24 hours after. Bone marrow-derived macrophages (BMDMs) were isolated by flushing the femurs and tibias of 10–14-week-old WT or *Marco^-/-^* mice. Briefly, mice were euthanized, and femurs and tibias were harvested under sterile conditions. Bone marrow was flushed from the bones using a 27-gauge needle and PBS. The cell suspension was filtered through a 70 μm cell strainer, centrifuged at 300 × g for 5 minutes, and resuspended in complete medium (RPMI 1640 medium supplemented with 10% fetal bovine serum (FBS) and 1% penicillin/streptomycin (P/S)). Then, BMDMs were plated at a density of 1 × 10⁶ cells/mL in non-tissue culture-treated Petri dishes and differentiated into MΦ macrophages by incubating them at 37ºC in complete RPMI 1640 Medium (Gibco) supplemented with M-CSF (30 ng/mL) (Biotechne, 416-ML). At day 7, BMDMs were harvested and cultured. For co-culture, 600,000 KCs or 1,000,000 BMDM cells/well were seeded in 6-well-plates (Corning).

For the proteomic studies, firstly, 80-90% confluent CCA SB1 cell lines^17^ cultured in DMEM 10% FBS 1% P/S were harvested by the addition of 0.05% trypsin-EDTA (Gibco – Thermo Fisher Scientific), centrifuged at 5,000 rpm for 5 minutes and resuspended in RPMI 10% FBS 1% P/S media. Then, 100,000 SB1 CCA cells were cultured on 8.0 µm pore size transwell membrane inserts (Corning® Costar® Transwell®) and placed in the top of the KCs or BMDMs. After 24 hours, both KCs/BMDMs and SB1 CCA cells were harvested for proteomics analysis. KCs and BMDMs were harvested in enzyme-free cell dissociation solution (Millipore) while SB1 CCA cells were harvested in radio-immunoprecipitation assay (RIPA) lysis buffer assisted by scraping. RIPA buffer contained: 150 mM NaCl, 50 mM Tris pH 7.5, 0.1% SDS, 1% Triton X100, 0.5% sodium deoxycholate, protease inhibitors (1 tablet / 50 ml, Complete; Roche) and phosphatase inhibitors (1 mM orthovanadate, 10 mM NaF, 100 mM β-glycerophosphate) (all from Sigma-Aldrich). Whole-cell lysates were collected and frozen at -80ºC to contribute to cell membrane disruption.

For the migration assay, after 48 hours of co-culture, inserts were transferred to a new 6-well plate, washed with 1× PBS, and stained by using a crystal violet solution (0.5% crystal violet, 4% PFA in PBS) for 20 minutes. After incubation, the inner surface of the insert was gently cleaned with a cotton swab to remove non-migrated cells. Images were taken using the Axio observer 7 microscope, and quantified as migrated area/total area.

**Mass spectrometry and proteomic analyses**

All samples were extracted or eluted using 7 M urea, 2 M thiourea, 4% CHAPS. Samples were incubated for 30 minutes at room temperature under agitation and digested following the Filter-Aided Sample Preparation protocol^21^ with minor modifications. Trypsin was added to a trypsin:protein ratio of 1:10, and the mixture was incubated overnight at 37ºC, dried out in a RVC2 25 speedvac concentrator (Christ), and resuspended in 0.1% formic acid (FA).

Samples were analyzed in a novel hybrid trapped ion mobility spectrometry–quadrupole time of flight mass spectrometer parallel accumulation serial fragmentation (tims TOF Pro with PASEF, Bruker Daltonics) coupled online to a nanoElute liquid chromatograph (Bruker). This mass spectrometer takes advantage of a novel scan mode termed parallel accumulation – serial fragmentation (PASEF), which multiplies the sequencing speed without any loss in sensitivity ^22^ and has been proven to provide outstanding analytical speed and sensibility for proteomics analyses.^23^ Sample (200 ng) was directly loaded in a 15 cm nanoElute FIFTEEN C18 analytical column (Bruker) and resolved at 400 nl/minute with a 30 minute gradient. The column was heated to 50ºC using an oven.

Spectral counts for each protein, in other words, the number of identified spectra matching to peptides from that protein (also named SpC or PSMs) were used for the differential analysis. Data were processed (log_2_ transformation, imputation) in the Perseus platform and a *t*-test was applied in order to determine the statistical significance of the detected differences. Heatmaps were generated using Heatmapper for data visualization^24^ and functional analyses of proteins were performed by gene ontology (GO) enrichment using DAVID Bioinformatics Resources.^25^

**THP-1 differentiation and polarization**

THP-1 monocytes were differentiated into macrophages by incubating with RPMI media supplemented with 10% FBS 1% P/S and 20 ng/ml Phorbol12-myristate 13-acetate (PMA) (Sigma-Aldrich) during 4 days (fresh media was added in the third day), followed by 24 hours incubation in RPMI culture medium containing 10% and 1% P/S. Subsequently, THP-1-derived macrophages were polarized into M1-like pro-inflammatory macrophages by the addition of 20 ng/ml IFN-γ (R&D) and 10 pg/ml lipopolysaccharide (LPS) (Sigma-Aldrich) to RPMI 10% FBS 1% P/S media during 24 hours, or into M2-like anti-inflammatory macrophages by incubating with 20 ng/ml IL-4 (R&D) and 20 ng/ml IL-13 (R&D) during 72 hours.

**RNA isolation and quantitative real time polymerase chain reaction (qRT-PCR) analysis**

Total RNA was isolated from liver tissue, by homogenizing small tissue samples (~20 mg), or cells in culture in 1 ml of Tri-reagent® (Sigma-Aldrich). Samples were subjected to a freeze-thaw cycle at -80ºC to facilitate digestion. Subsequently, 200 µl of chloroform (Merck) was added to the samples and, after vortexing, samples were incubated for 15 minutes at RT and centrifuged for another 15 minutes at 18,000 g at 4ºC. For RNA precipitation, the upper phase was transferred into a new tube and 500 µl of 2-propanol (AppliChem Panreac) was added. Samples were mixed by inverting them several times, incubated 10 minutes at RT and centrifuged for 15 minutes at 18,000 g at 4ºC. Once the RNA pellet was formed, supernatant was discarded and 1 ml of 75% ethanol was added to the samples and were vortexed briefly in order to wash the pellets. Samples were centrifuged for 15 minutes at 18,000 g at 4ºC, supernatant was discarded and pellets were left to dry on ice. Finally, RNA pellets were resuspended in DNase/RNase-free distilled water. RNA quantification was carried out with NanoDrop® ND-1000 apparatus (Thermo-Fisher Scientific).

The RT of RNA extracted from primary isolated mouse cells was carried out using the SuperScript® VILO™ cDNA Synthesis Kit (Life Technologies) following manufacturer’s instructions. Briefly, 2 μl of 10X SuperScript® Enzyme Mix, 4 μl of 5X VILO™ Reaction Mix and DNase/RNase-free distilled water to a total volume of 20 μl was added to 500 ng of total RNA of each sample. RT was performed in a Verity 96 well thermal cycler (Applied Biosystems) using a 3-step protocol: i) 10 minutes at 25ºC, ii) 1 hour at 42ºC, and iii) 5 minutes at 85ºC. DNase/RNase-free distilled water was added to the newly synthetized complementary DNA (cDNA) to obtain a final concentration of 12.5 ng/μl.

The RT of RNA derived from mouse liver tissue and cells in culture was performed on 1 μg of extracted RNA. First, a DNase incubation to remove genomic DNA was carried out adding 1 μl of DNase I Amplification Grade (Invitrogen) and 1 μl of 10X DNase I Reaction Buffer (Invitrogen) during 20 minutes at 37ºC. Next, 1 μl of 25 mM ethylenediaminetetraacetic acid (EDTA) (Invitrogen) was added to chelate magnesium and stop DNase activity. Samples were incubated for 10 minutes at 65°C, 1 minute at 90°C and kept at 4°C. The cDNA synthesis step was performed by adding 30 μl of RT Mix to the samples. The mix included 8 μl Buffer 5X, 4 μl Random primers 100 ng/μl, 4 μl Deoxy-nucleotide-triphosphate mix (dNTPs), 2 μl 1,4-dithiothreitol (DTT), 1.2 μl RNase OUT, 1.2 μl M-MLVRT (all from Invitrogen) and 9.6 μl DNase/RNase-free distilled water. The reaction was achieved by incubating the samples for 60 minutes at 37°C, 1 minute at 95°C and cooled at 4°C. Finally, the newly synthetized cDNA was diluted to a final concentration of 12.5 ng/μl with DNase/RNase-free distilled water.

Gene expression was analyzed by the addition of a mix containing 10 μl of iQ™ SYBR® Green Supermix (Bio-Rad), 0.6 μl of 10 μM appropriate forward and reverse primers (**Supplementary Table 4**), and DNase/RNase-free distilled water to a final volume of 17 μl per sample. After placing the mix in Hard-Shell 96-well PCR plates (Bio-Rad), 3 µl of the desired cDNA sample at 12.5 ng/μl were added to each well. Afterwards, the plate was sealed with Microseal “B” seal (Bio-Rad) and the RT-qPCR reaction was carried out in a CFX 96 apparatus (Bio-Rad) following an iQ™ SYBR® Green Supermix standard protocol. Briefly, cDNA denaturation and enzyme activation were achieved by an initial step of 10 minutes at 95°C. Then, for DNA amplification 40 cycles of 3 steps including 15 seconds at 95ºC for DNA denaturation, 30 seconds at 60ºC for primer binding and 45 seconds for 72ºC for extension were performed. A dissociation curve to confirm primer specificity was obtained by gradually increasing the temperature from 60°C to 93ºC. Expression levels of glyceraldehyde-3-phosphate dehydrogenase (GAPDH) or hypoxanthine phosphoribosyltransferase 1 (HPRT1) were used as housekeeping control. Desired gene expression was determined using the ΔCT calculation. mRNA levels are indicated as arbitrary units (AU) or relative to the control condition.

**Statistical analysis**

The statistical analysis was performed employing the GraphPad Prism 8 (GraphPad Software) or R software (version 4.0.2; R Foundation for Statistical Computing, Vienna, Austria). Once the normal distribution of the data was assessed with Shapiro-Wilk test, parametric tests were employed for normally distributed and corresponding non-parametric tests for non-normally distributed data. When comparing two independent groups, parametric two-tailed Student’s *t*-test or non-parametric Mann Whitney test were employed, unless when specified. For comparisons between more than two data sets, parametric one-way analysis of variance (ANOVA) with Tukey’s *post hoc* test or non-parametric Kruskal-Wallis test followed by Dunn’s multiple comparison test were used. Adjusted p-value was obtained employing the False Discovery Rate (FDR) method. For correlation analysis parametric Pearson’s test or non-parametric Spearman’s correlation test were implemented. In order to compare survival Kaplan-Meier curves, Log rank (Mantel-Cox) statistical test was employed and a *p*-value <0.05 was used as a cut-off to detect significant differences. Data are indicated as mean ± standard error of the mean (SEM). Statistically significant data is represented in figures by *. *, **, *** and **** denote a *p-*value of ˂0.05, ˂0.01, ˂ 0.001 and < 0.0001, respectively. In correlation analysis, correlation coefficient and exact *p*-values are shown.

Figure S1.

**
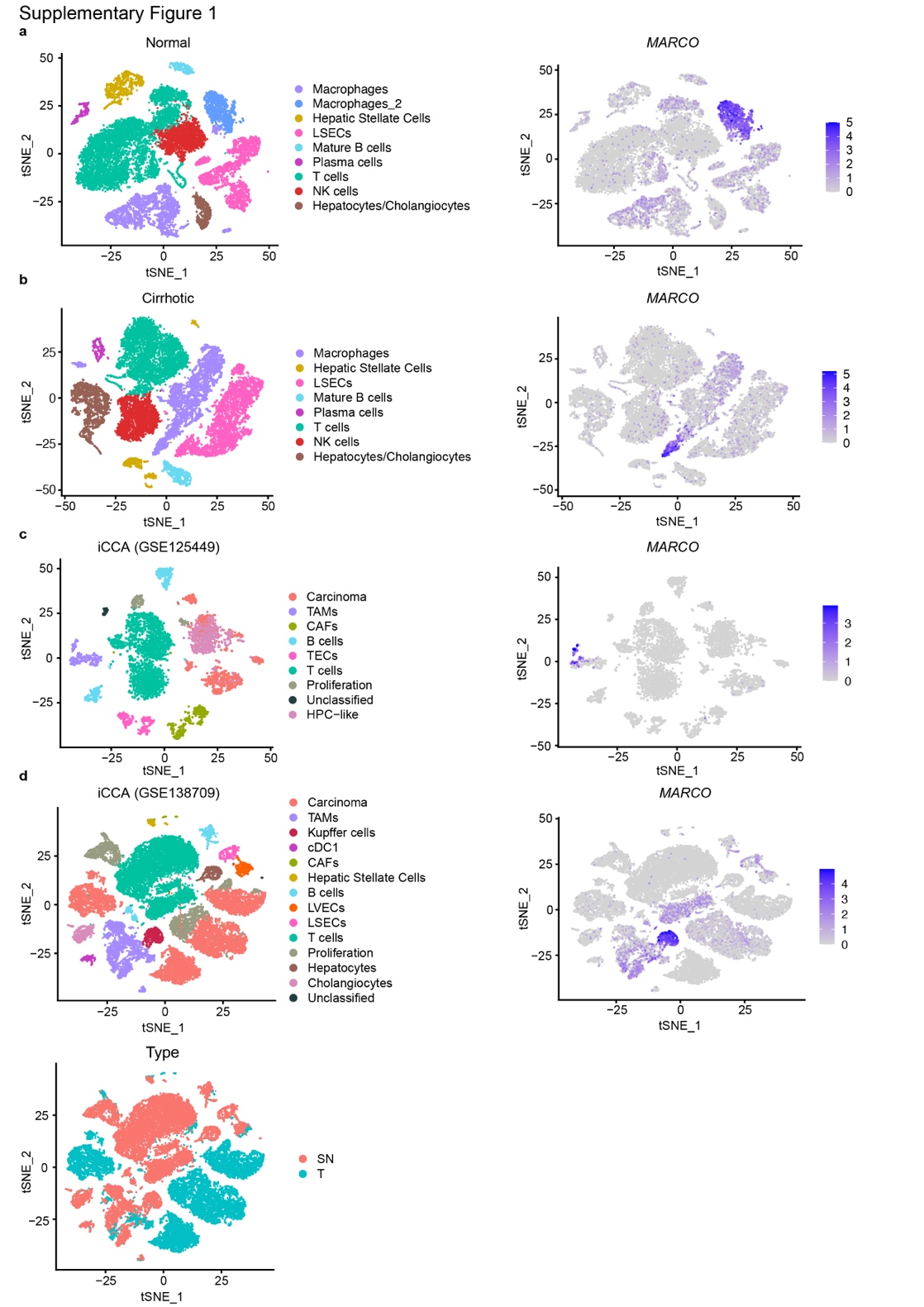
**

**Supplementary Fig. 1. Analysis of cell type-specific *MARCO* expression in normal and cirrhotic livers and iCCA tumors by single-cell RNA sequencing.** Left: graph-based clustering using t-distributed Stochastic Neighbour Embedding (t-SNE) projection depicting cellular composition of different human samples. Cells that share similar transcriptome profiles are grouped by colours and were annotated using lineage specific markers. Right: t-SNE plot depicting *MARCO* expression in macrophages in (**a**) healthy (n=5) and (**b**) cirrhotic livers (n=5) and TAMs in different cohorts of iCCA tumors (**c**) [GSE125449 (n=10)] and (**d**) GSE138709 (n= 4T, 3SL). Abbreviations; CAF, cancer-associated fibroblast; DC, dendritic cell; HEP, hepatic progenitor cell; iCCA, intrahepatic cholangiocarcinoma; LSEC, liver sinusoidal endothelial cells; LVEC, liver vascular endothelial cell; MARCO, macrophage receptor with collagenous structure; NK, natural killer; SL; surrounding liver; T, tumor; TAM, tumor-associated macrophage; TEC, tumor endothelial cell; tSNE, t-distributed stochastic neighbour embedding.

**Figure S2.**


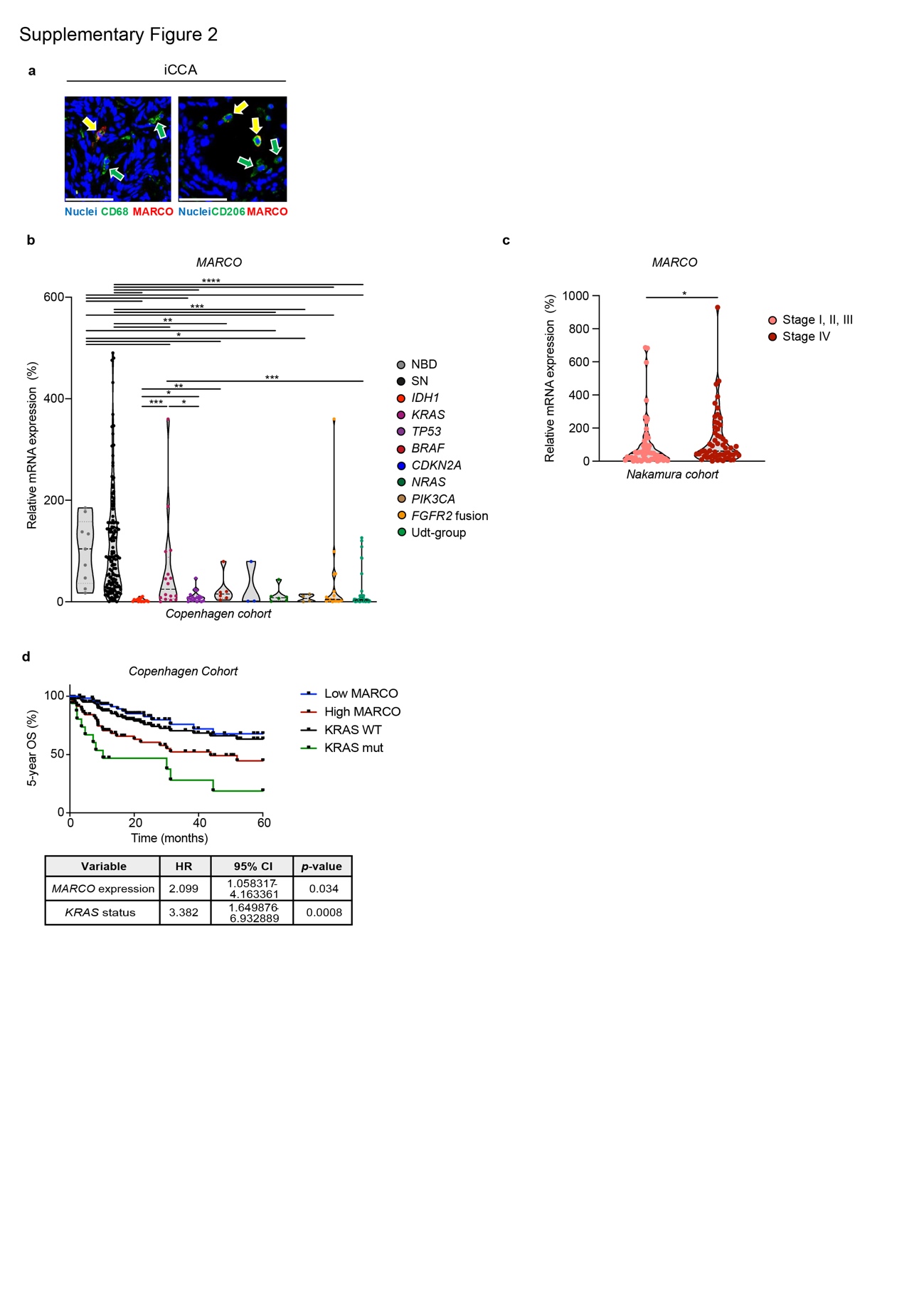


Supplementary Fig. 2. Analysis of *MARCO* expression in non-tumoral surrounding and tumoral tissue, and association of its expression to patients’ outcome. (a) Double immunofluorescence staining showing the expression of MARCO in a subset of CD68^+^ or CD206^+^ TAMs in iCCA. Scale Bars= 65um. (b) *MARCO* mRNA (microarray) expression in iCCA tumors harbouring *IDH1* (n=16), *KRAS* (n=16), *TP53* (n=17), *BRAF* (n=6), *CDKN2A* (n=3), *NRAS* (n=6), or *PIK3CA* (n=4) mutations, *FGFR2* fusions (n=18) or harbouring no alterations in any of these genes, Udt-group (n=64) (Copenhagen cohort). (c) *MARCO* mRNA (microarray) expression in iCCA tumors grouped by tumor stage, according to AJCC guidelines in the Nakamura cohort: early- [stage I, II and III (n=53)] or late- [stage IV (n=57)] stage. (d) 5-year overall survival curves of patients with iCCA from the Copenhagen cohort (n=120). Red and blue lines indicate patients with iCCA classified according to high *MARCO* vs low *MARCO* (at or below median) expression, while black and green lines indicate patients with iCCA according to *KRAS* mutation status (WT or *KRAS* mutated). Multivariate Cox analysis was performed. (b,c) Unpaired t-test and Mann-Whitney test were employed. Data are shown as mean ± SEM. *, **, *** and **** denote *p*-values of <0.05, <0.01, <0.001 and <0.0001, respectively. Abbreviations; iCCA, intrahepatic cholangiocarcinoma; MARCO, macrophage receptor with collagenous structure; NBD, normal bile duct; SN, surrounding normal; Udt, undetermined.

Figure S3.


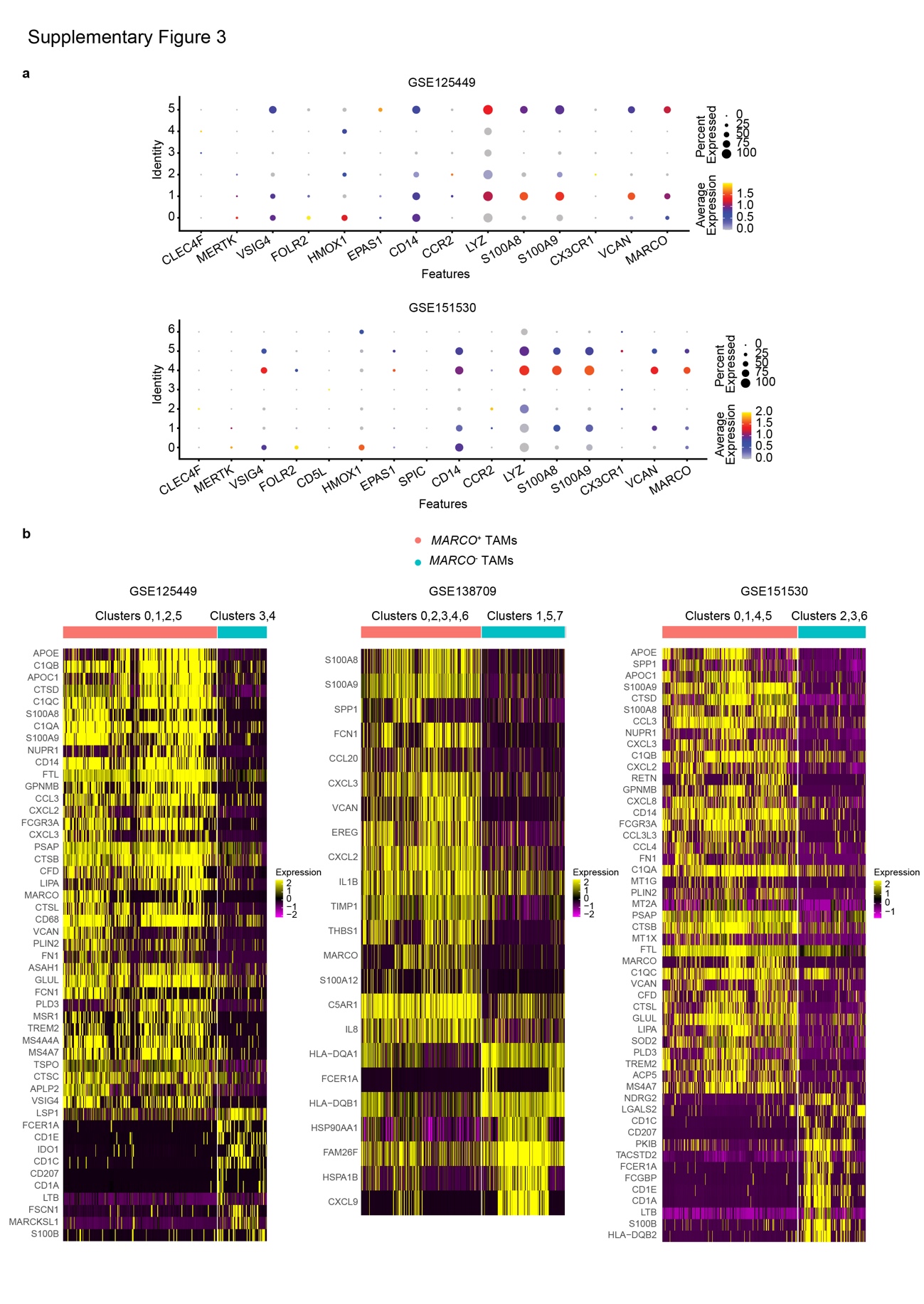


**Supplementary Fig. 3. Analysis of *MARCO* expression in TAMs by single-cell RNA sequencing.** (**a**) Expression of key Kupffer cell and monocytic markers in the identified TAM clusters in GSE125449 and GSE151530. (**b**) Heatmap of differentially expressed genes (|log2FC|<1.5, adjusted *p*-value <0.01) between *MARCO*-expressing and non-expressing TAMs in human iCCA. Abbreviations; iCCA, intrahepatic cholangiocarcinoma; MARCO, macrophage receptor with collagenous structure; TAM, tumor-associated macrophage.

Figure S4.

**
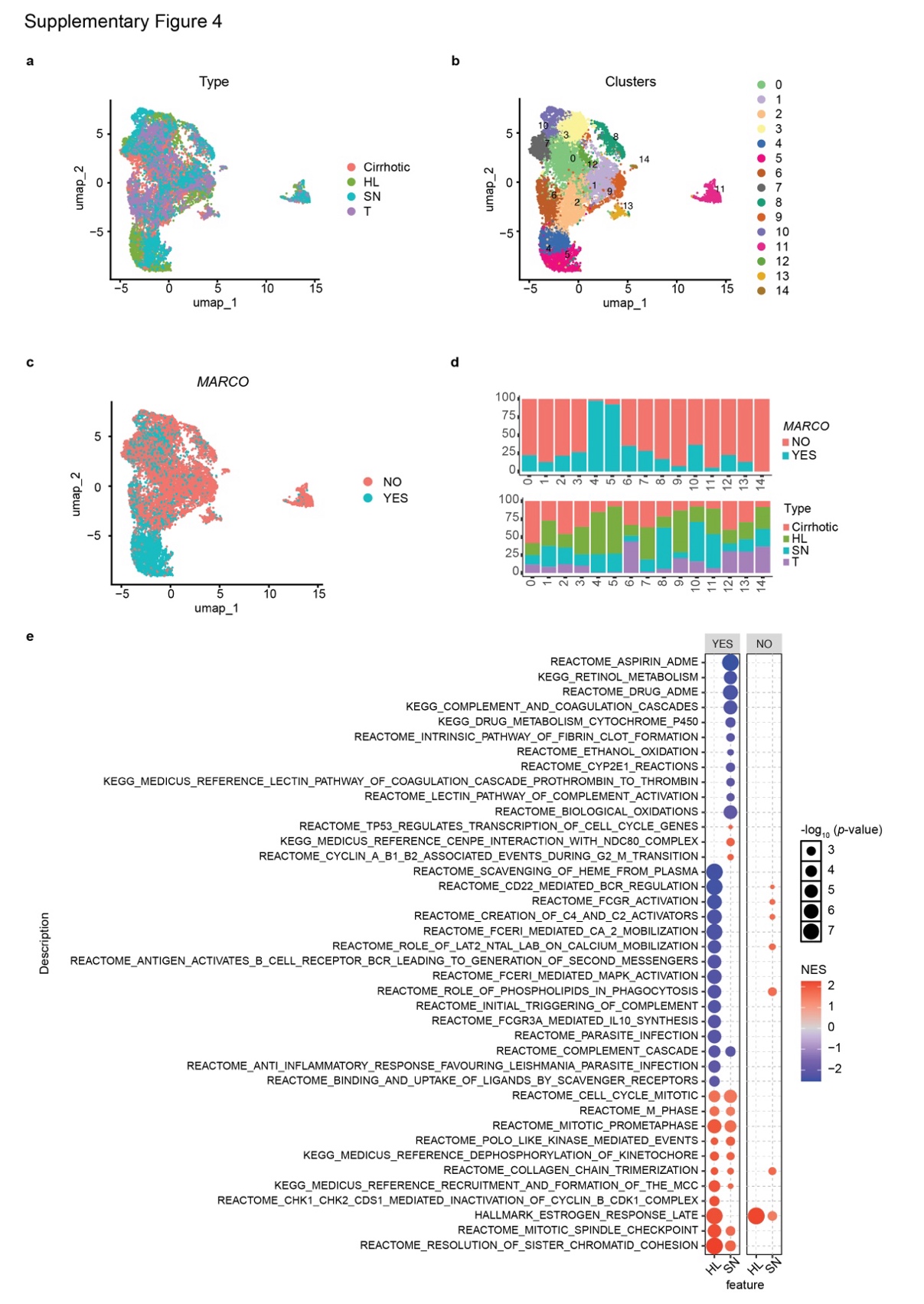
**

**Supplementary Fig. 4. Characterization of *MARCO*^+^ TAMs compared to *MARCO*^+^ macrophages in healthy and surrounding liver tissue. (a)** UMAP visualization of integrated macrophage populations. Liver focused single cell studies were merged (GSE125449, GSE138709, GSE151530, GSE146409, GSE136103, GSE149614). **(b)** Clustering of macrophage populations across conditions. **(c)** Feature plot showing *MARCO* expression across macrophage clusters. **(d)** Bar plot of *MARCO* expression across macrophage clusters. **(e)** GSEA of differentially expressed genes comparing *MARCO*^+^ (YES) and *MARCO*^-^ (NO) TAMs with *MARCO*^+^ (YES) and *MARCO*^-^ (NO) macrophages from SN and HL. Reactome and KEGG pathways were selected based on adjusted *p*-value <0.01 and |NES| > 0.2. The top five up- and downregulated pathways are shown for each comparison group (HL, SN and both vs Tumor). Abbreviations: HL, healthy liver; SN, surrounding normal; T, tumor.

Figure S5.

**
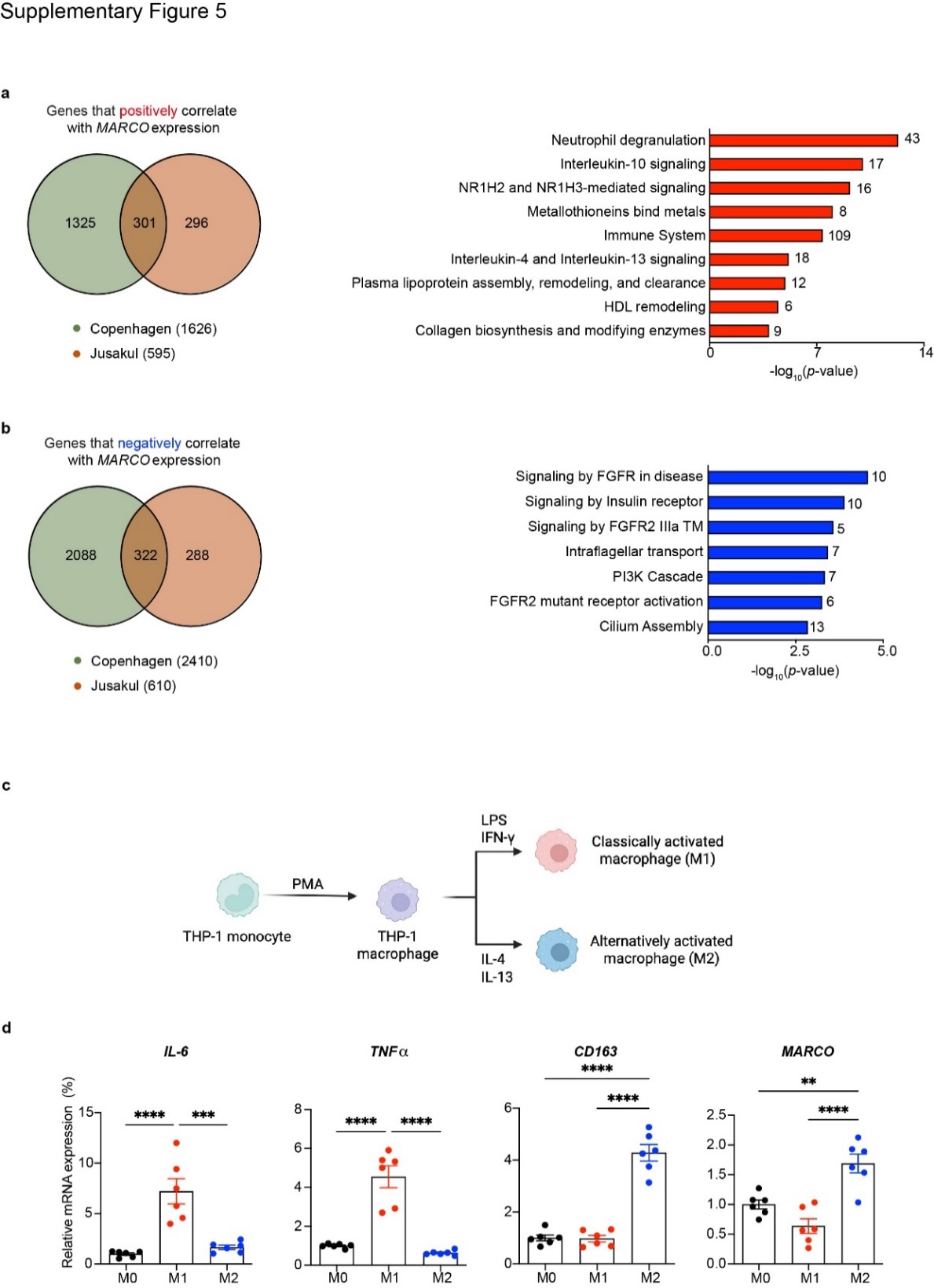
**

**Supplementary Fig. 5. Correlation analysis of MARCO expression in iCCA and functional characterization in macrophages.** (**a**) Venn diagram of genes that positively correlate with *MARCO* mRNA expression in tumors from both Copenhagen and Jusakul cohorts, and Reactome analysis of the entities related to the common genes (n=301 genes). (**b**) Venn diagram with the genes that negatively correlate with *MARCO* mRNA expression in tumors from both Copenhagen and Jusakul cohorts of patients, and Reactome analysis of the entities related to the common genes (n=322 genes). (**c**) Schematic representation of the experiment. (**d**) mRNA expression of pro-inflammatory macrophage (M1 phenotype) markers *IL-6* and *TNFα*, anti-inflammatory macrophage (M2 phenotype) marker *CD163*, and *MARCO* in M1 and M2 differentiated THP-1-derived macrophages (n=6). One way ANOVA test was used. Data represent mean ± SEM and **, *** and **** denote a *p*-value of <0.01, <0.001 and <0.0001, respectively. Abbreviations; iCCA, intrahepatic cholangiocarcinoma; IL, interleukin; MARCO, macrophage receptor with collagenous structure; TNF, tumor necrosis factor.


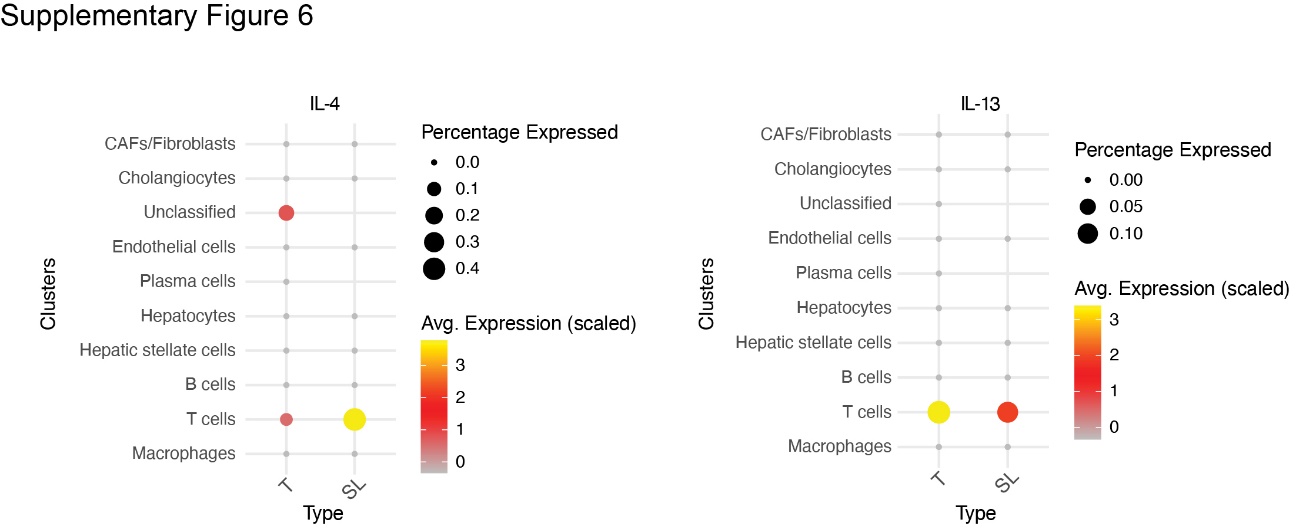
Figure S6.

**Supplementary Fig. 6.** **Cellular sources of IL-4 and IL-13 in intrahepatic cholangiocarcinoma (iCCA) based on scRNA-seq analysis.** Publicly available datasets from iCCA samples were analyzed to determine the cellular expression patterns of IL-4 and IL-13.

**Figure S7.**

**
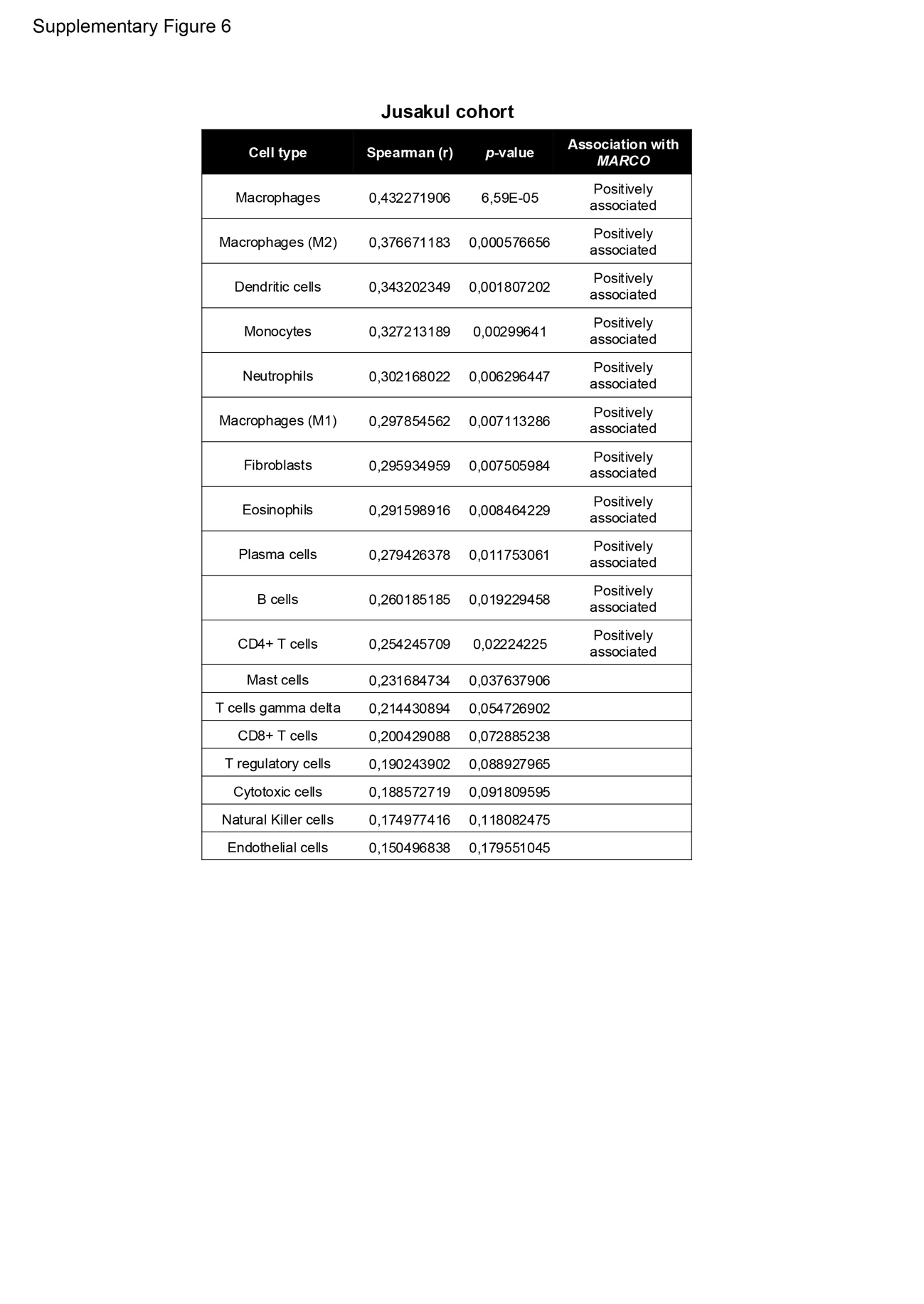
**

**Supplementary Fig. 7. Correlation of *MARCO* expression with bioinformatic estimates of specific ell types in human iCCA tumors employing ConsensusTME.** Association of *MARCO* expression with different cell types present in iCCA tumors from the Jusakul cohort of patients. Spearman’s correlation coefficient was used. Abbreviations; iCCA, intrahepatic cholangiocarcinoma; MARCO, macrophage receptor with collagenous structure.

**
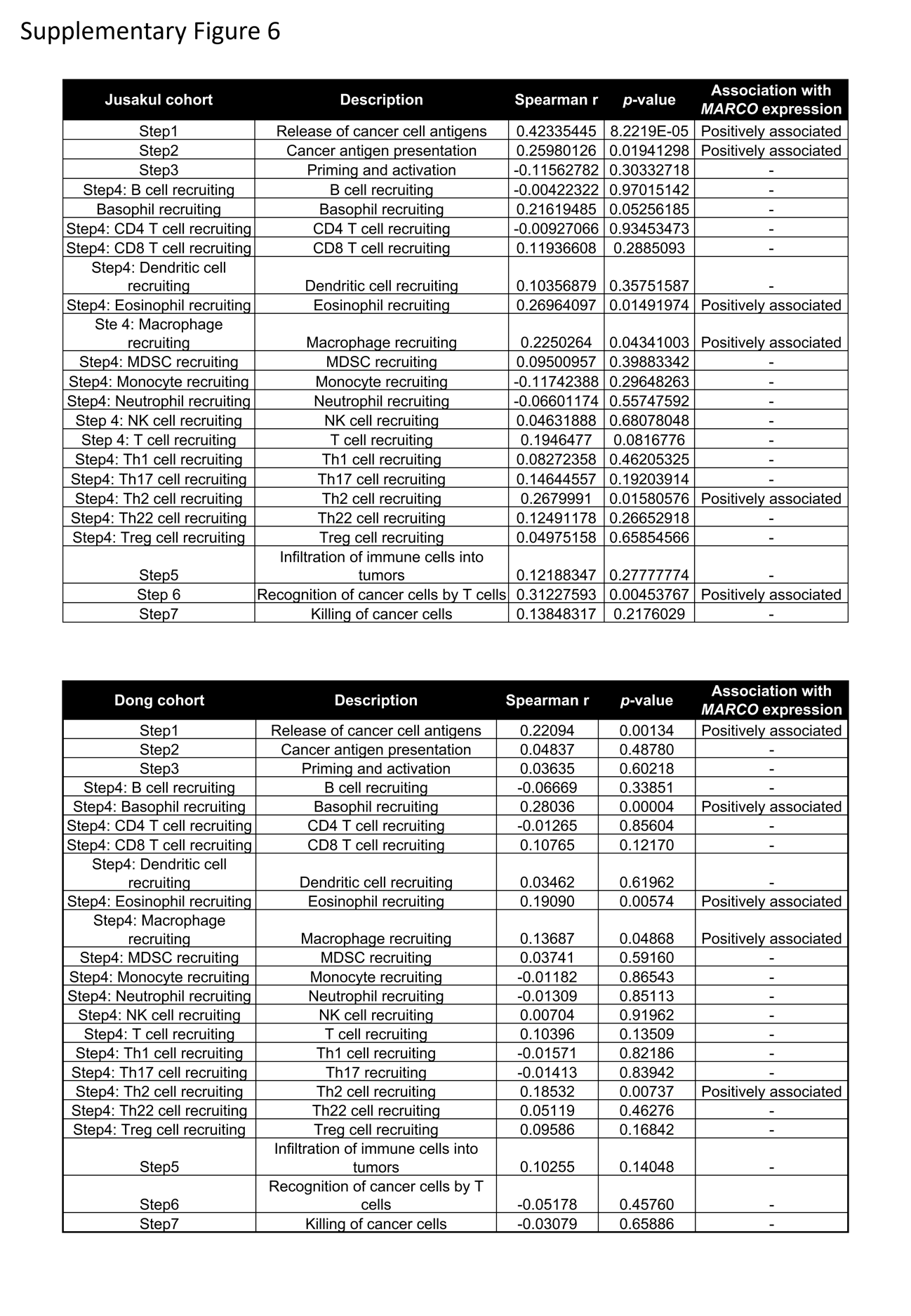
Figure S8.**

**Supplementary Fig. 8. Association of *MARCO* expression with gene expression signatures related to the different steps of the cancer immune cycle.** Association of *MARCO* expression with each of the 7 steps of the cancer immune cycle in the iCCA samples from the Jusakul and Dong cohorts of patients. The activity of the different stages of the cycle was estimated in the iCCA samples using the TIP tool. Spearman’s correlation coefficient was used. Abbreviations; iCCA, intrahepatic cholangiocarcinoma; MARCO, macrophage receptor with collagenous structure.

**Figure S9.**

**
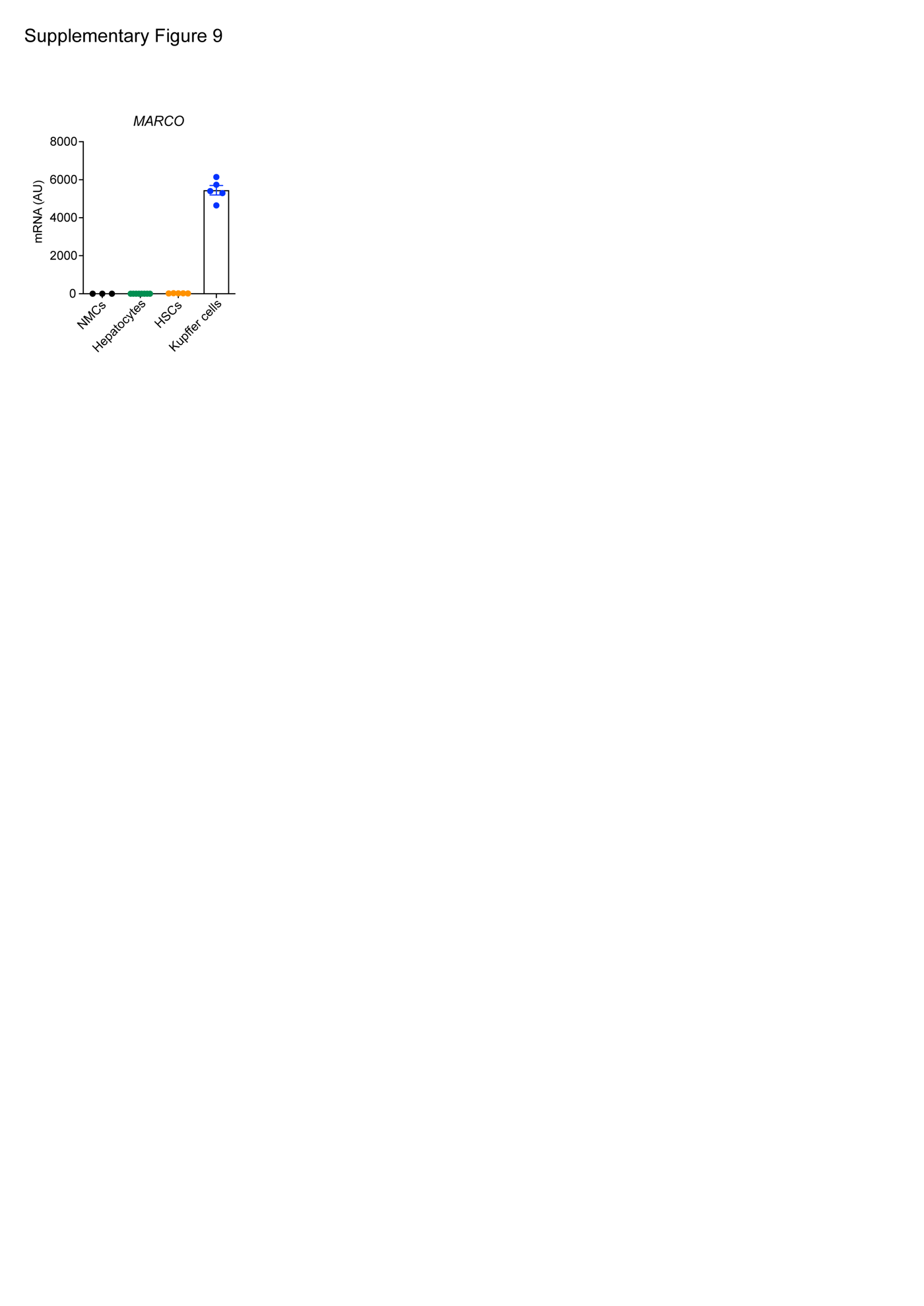
**

Supplementary Fig. 9. *Marco* expression in the different mouse liver cell populations. *Marco* mRNA expression in mouse cholangiocytes (n=5), hepatocytes (n=8), HSCs (n=5) and KCs (n=5). Data are shown as mean ± SEM. Abbreviations; AU, arbitrary units; HSCs, hepatic stellate cells; KCs, Kupffer cells; Marco, macrophage receptor with collagenous structure; NMC, normal mouse cholangiocytes.

**Figure S10.**


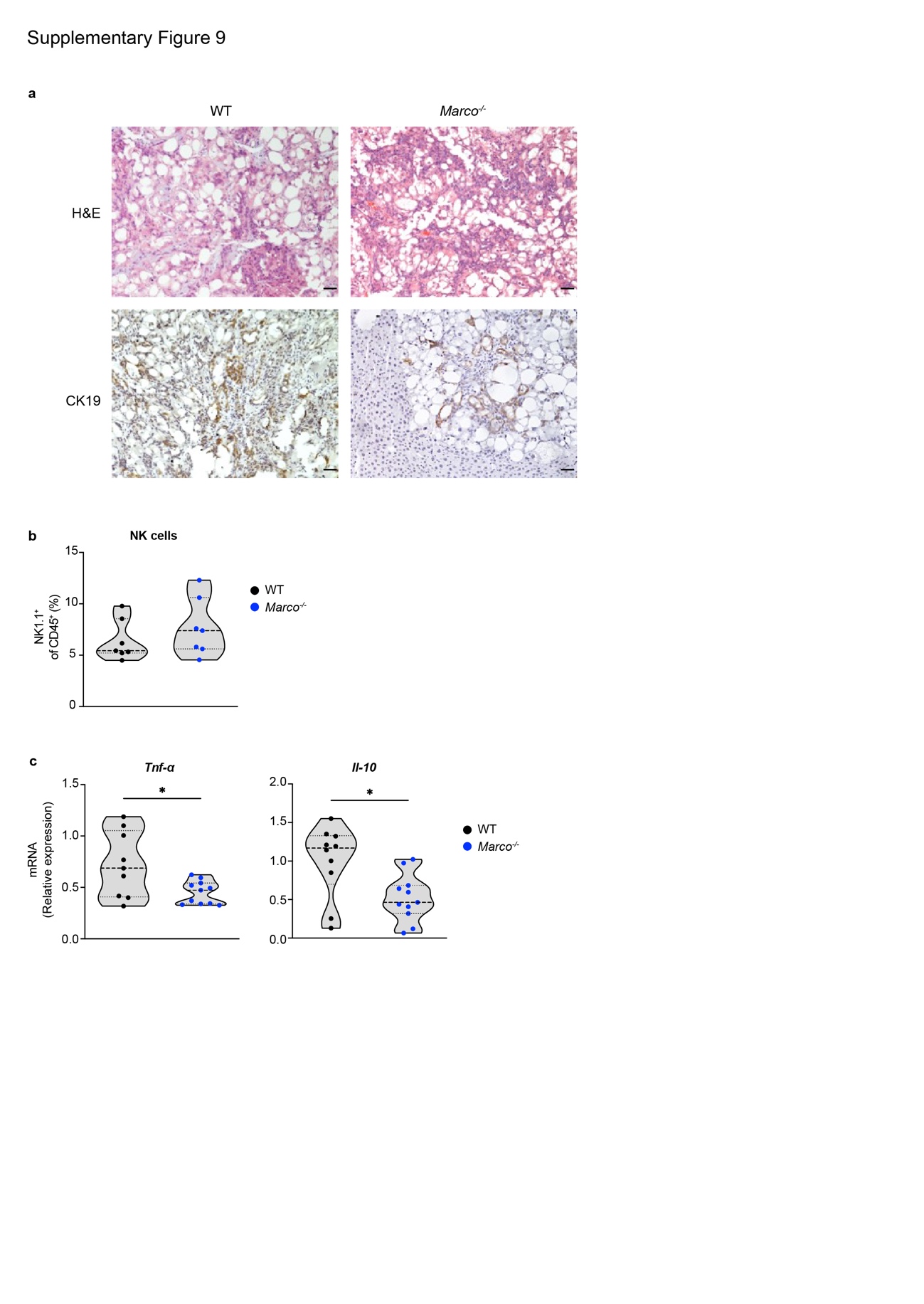


Supplementary Fig. 10. Characterisation of WT and *Marco*^-/-^ mice subjected to a murine model of iCCA based on constitutively active *AKT* and *YAP* overexpression. (a) H&E and CK-19 immunohistochemistry representative images of iCCA tumors in livers from WT and *Marco*^-/-^ mice (Scale bars: 50μm). (b) Percentage of NK cells among total immune cells (CD45^+^). (c) Hepatic mRNA expression of *Tnf-α* and *Il-10*. Data are shown as mean ± SEM. (B,C) Non-parametric Mann-Whitney t-test was employed. * denotes a *p*-value of <0.05. Abbreviations: CK19, cytokeratin 19; H&E, haematoxylin and eosin; Il, interleukin; Marco, macrophage receptor with collagenous structure; NK, natural killer; Tnf, tumor necrosis factor.

**Figure S11.**


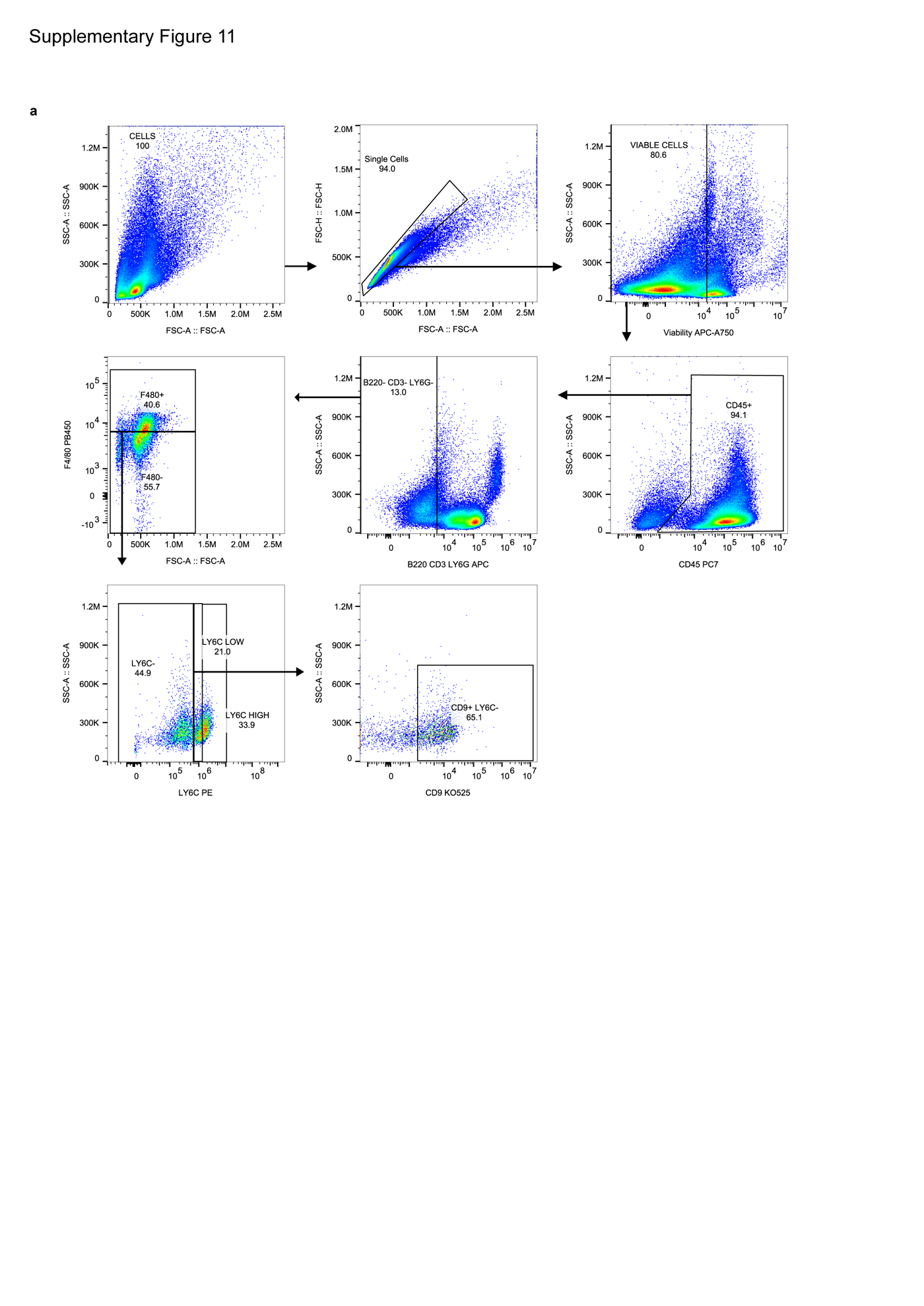


Supplementary Fig. 11. Gating strategy for CD9^+^ macrophages. Related to Figures 5, 6. CD9^+^ macrophages are gated as single cells, viable and CD45^+^B220^-^CD3^-^Ly6G^-^F4/80^+^Ly6C^-^CD9^+^.

**Figure S12.**


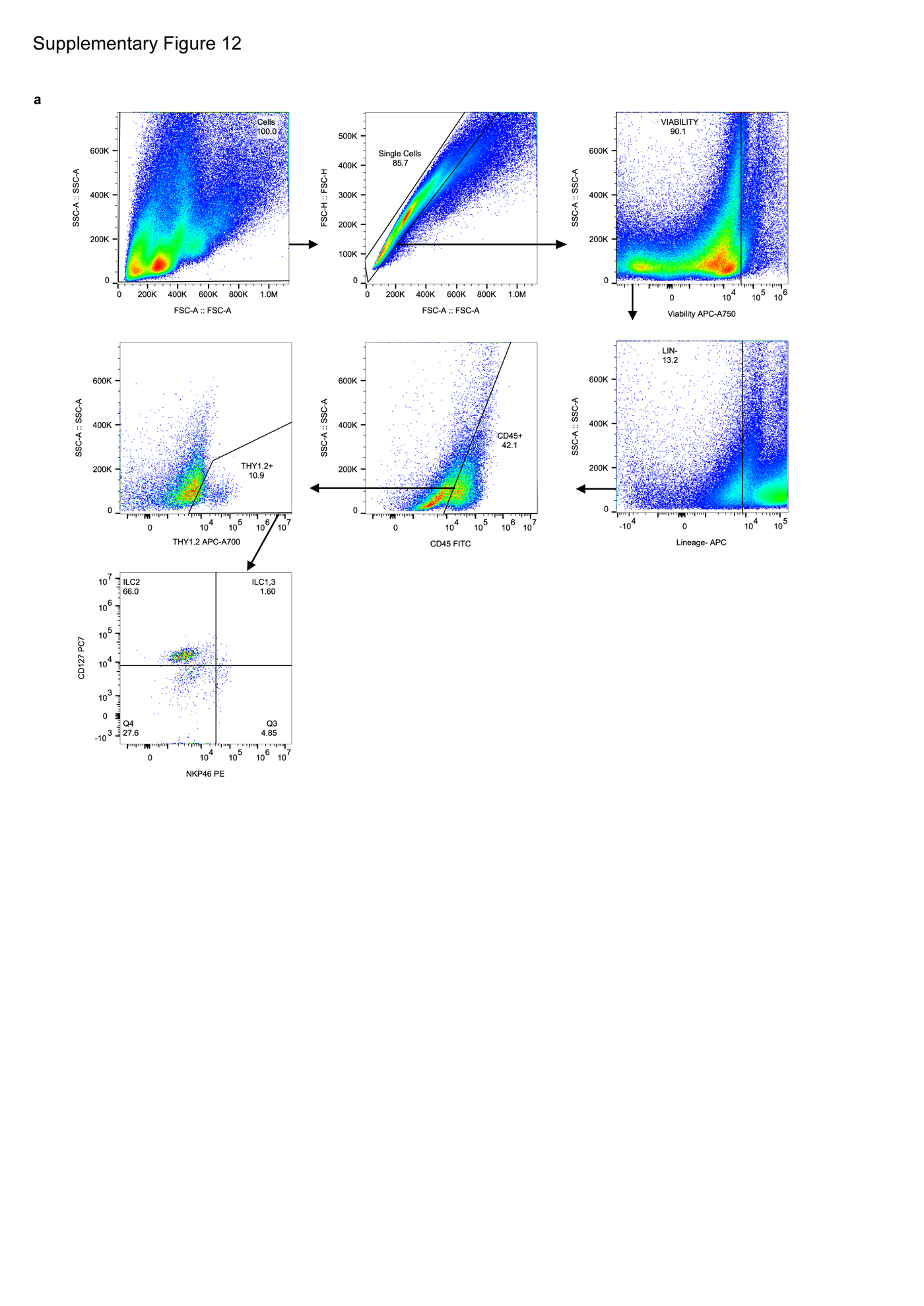


Supplementary Fig. 12. Gating strategy for ILC2 cells. Related to Figure 5. ILC2s are gated as single cells, viable, Lineage negative, CD45^+^Thy1.2^+^CD127^+^NKp46^-^ cells.

**Figure S13.**

**
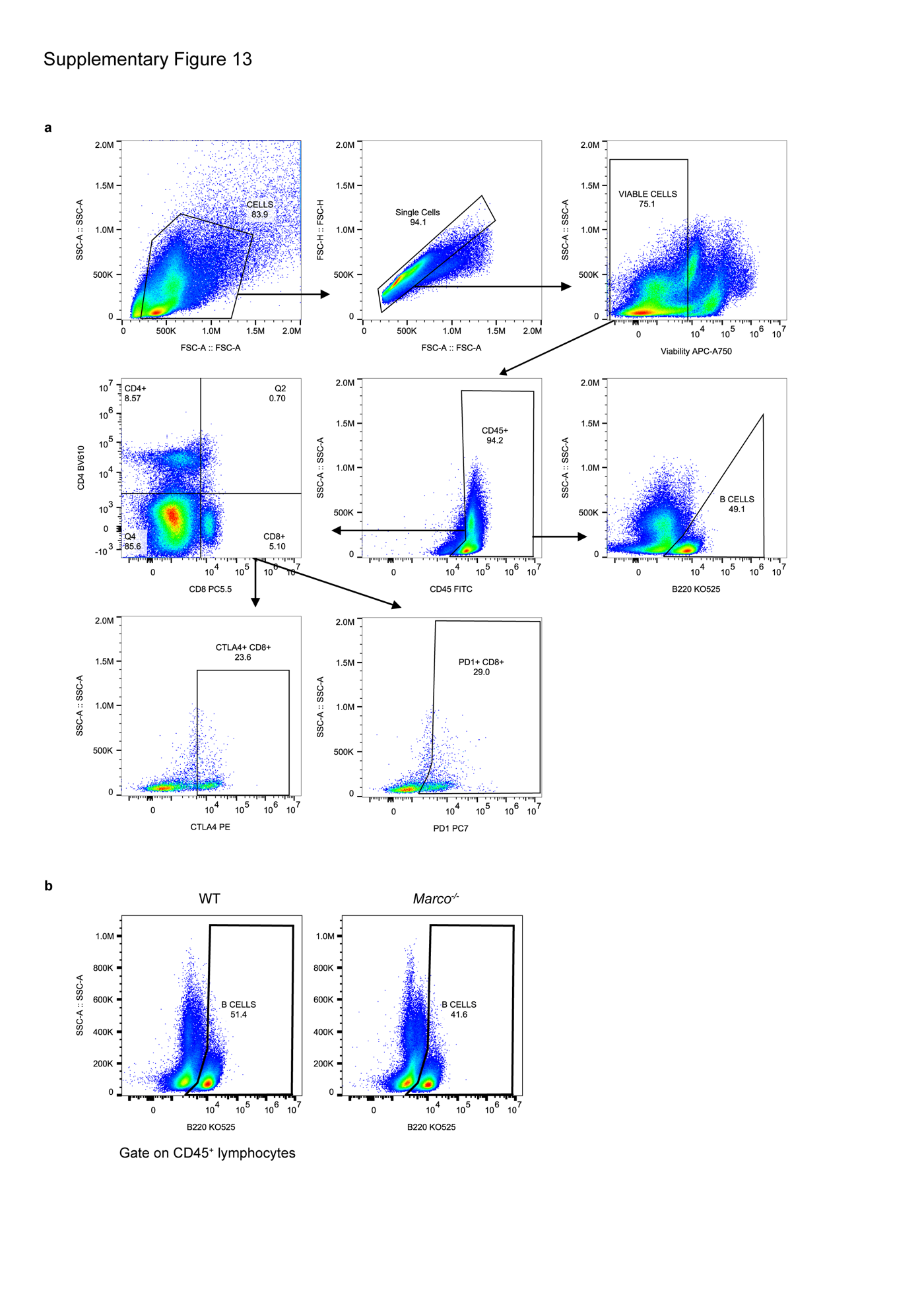
**

**Supplementary Fig. 13. Gating strategy for PD-1^+^ and CTLA-4^+^ cytotoxic T cells, and B cells.** Related to Figures 5, 6. (**a**) Cytotoxic T cells are gated as single cells, viable CD45^+^CD8^+^ cells. B cells are gated as single cells, viable and CD45^+^B220^+^. (**b**) Representative graph of B cell percentage in WT and *Marco*^-/-^ mice.

**Figure S14.**


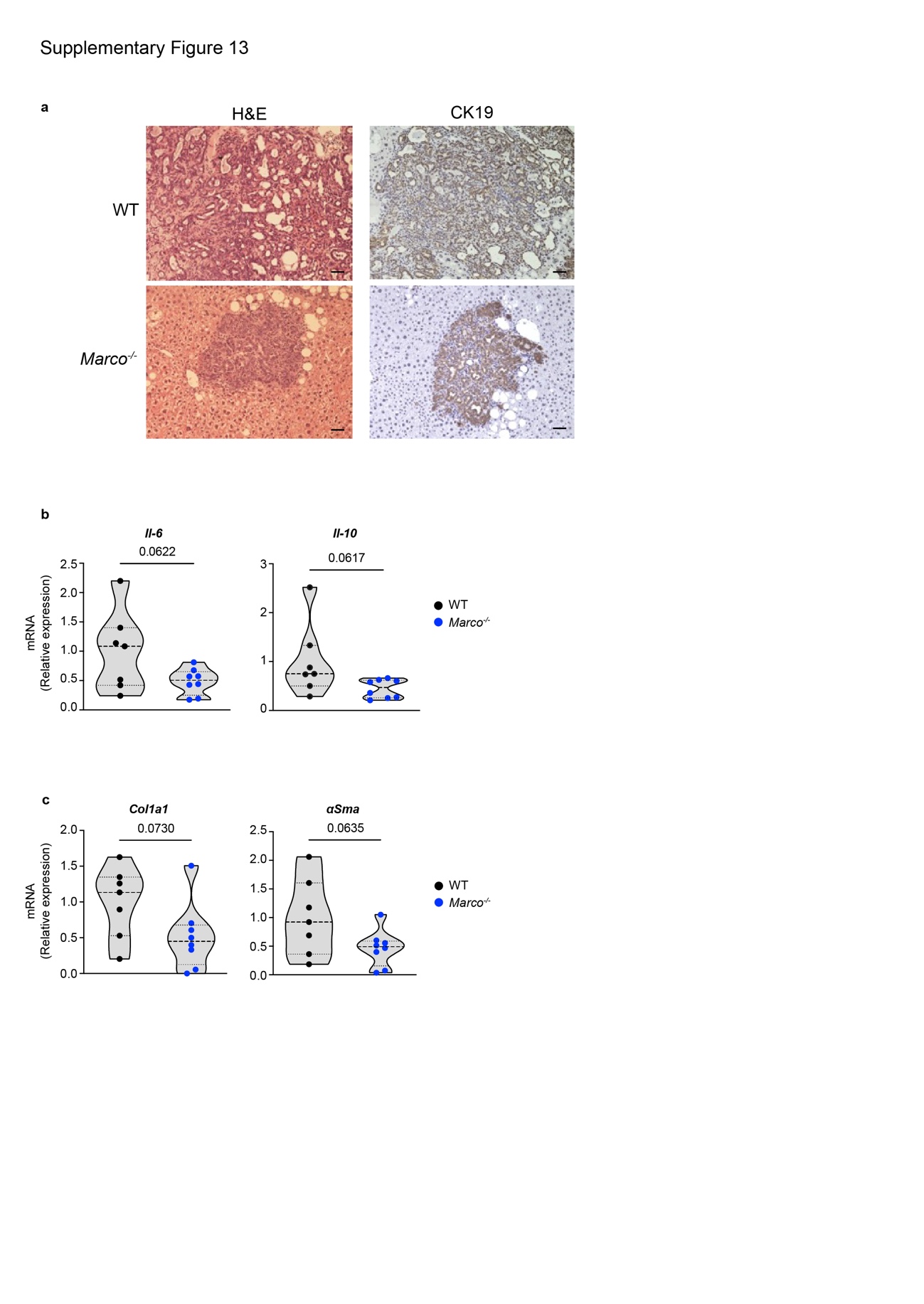


Supplementary Fig. 14. Characterisation of WT and *Marco*^-/-^ mice subjected to a murine model of iCCA based on the overexpression of constitutively active *AKT* and *Fbxw7ΔF* in the liver. (a) Representative images of iCCA tumors in livers from WT and *Marco*^-/-^ mice. (b) Hepatic mRNA expression levels of *Il-6, Il-10*, (c) *Col1a1* and *αSMA*. Data are shown as mean ± SEM. Parametric student’s t-test and non-parametric Mann-Whitney test were used. Abbreviations: αSMA, alpha-smooth muscle actin; Col1a1, alpha-1 type I collagen; CK, cytokeratin; H&E, haematoxylin and eosin; Il, interleukin; Marco, macrophage receptor with collagenous structure.

**Figure S15.**


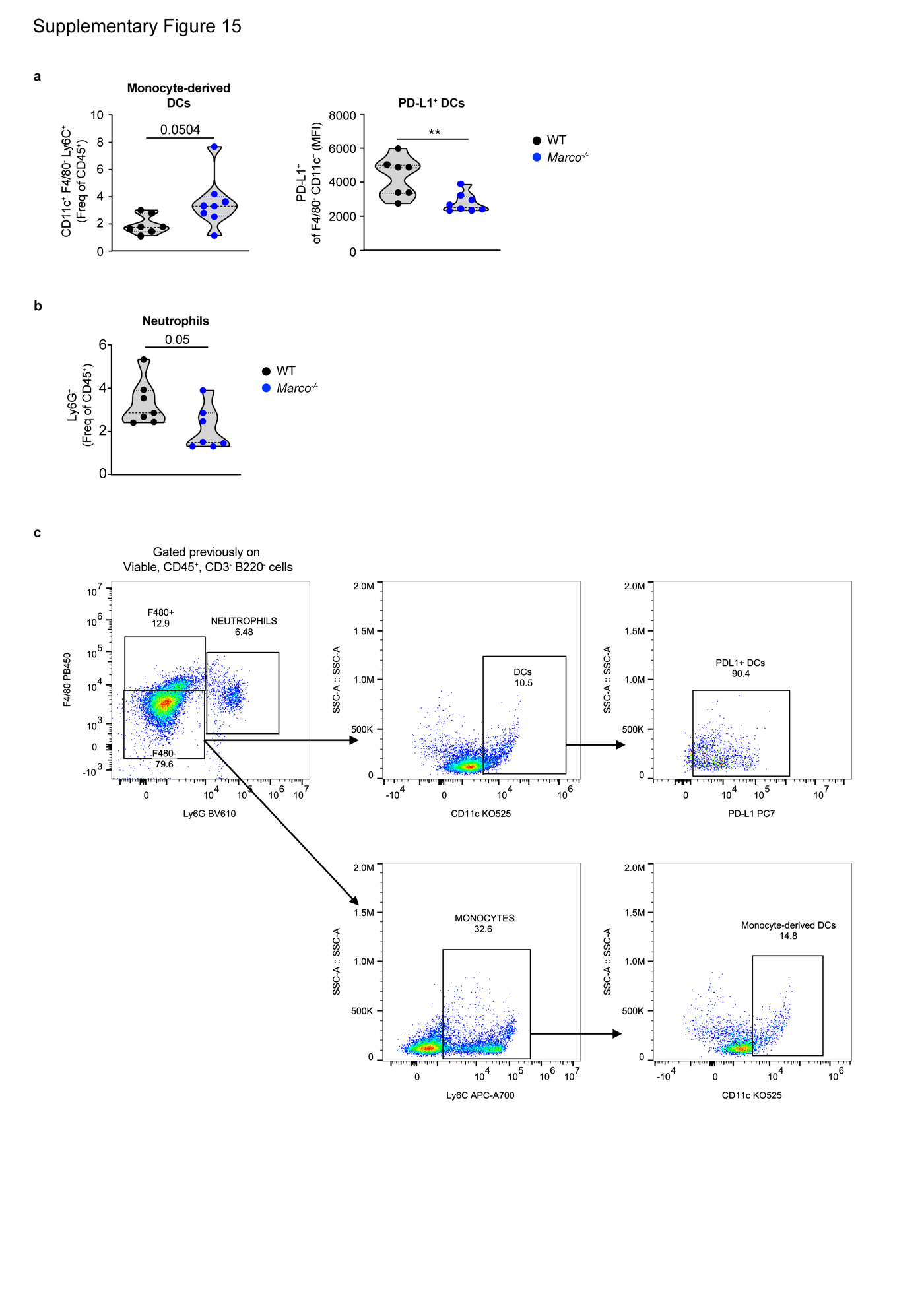
Supplementary Fig. 15. Myeloid compartment analysis of the livers of mice subjected to iCCA development by the overexpression of constitutively active *AKT* and *Fbxw7ΔF*. (a) Percentage of monocyte-derived DCs among total immune cells (CD45^+^), and MFI of PD-L1^+^ in DCs. (b) Neutrophil populations among total immune cells (CD45^+^). (c) Gating strategy of PD-L1^+^ DCs and monocyte-derived DCs. Unpaired t-test was employed. ** denotes a *p*-value of <0.01, respectively. Abbreviations: DCs, dendritic cells; Marco, macrophage receptor with collagenous structure; MFI mean fluorescence intensity.

**Figure S16.**


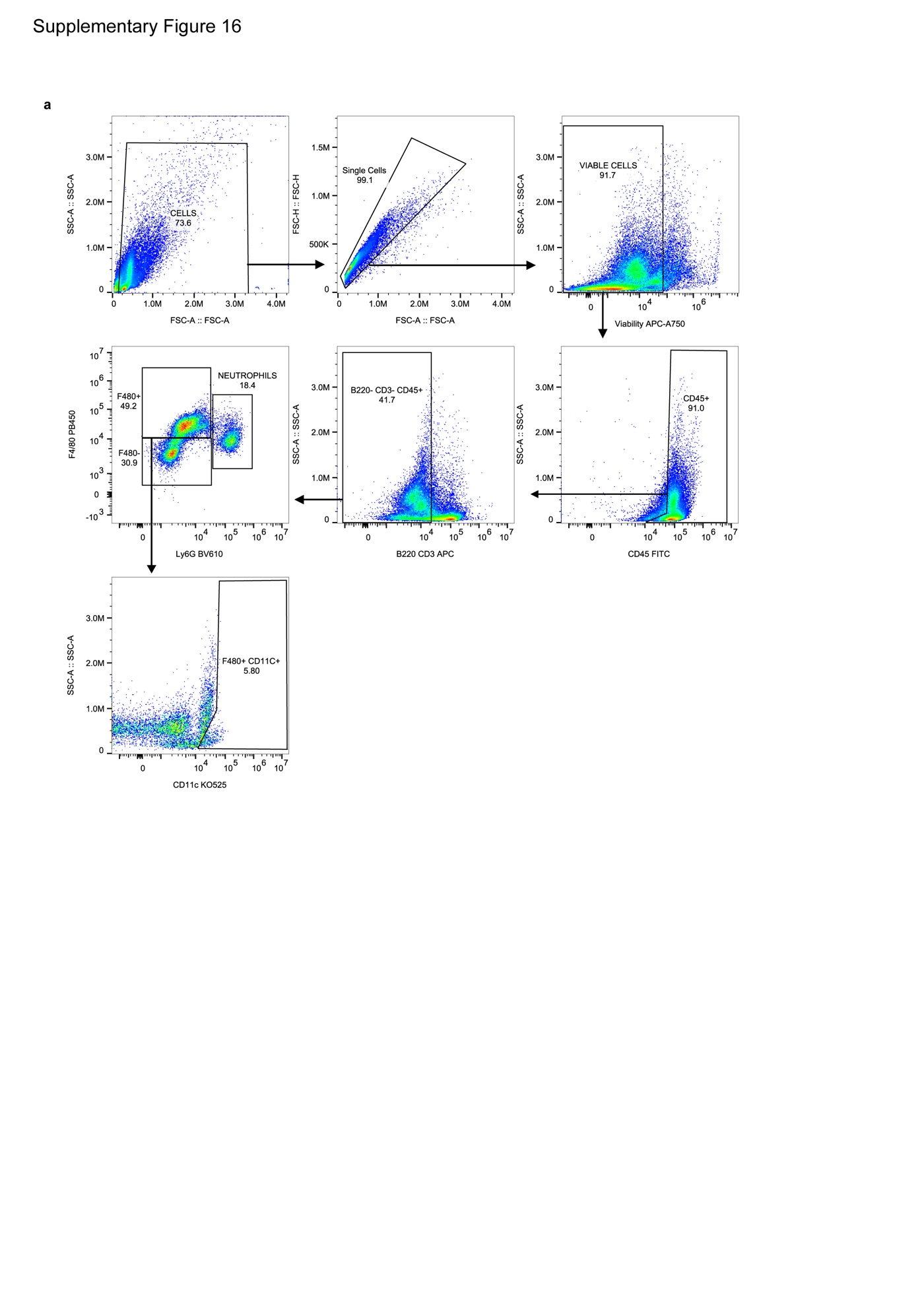


Supplementary Fig. 16. Gating strategy for CD11c^+^ macrophages. Related to Figure 6. CD11c^+^ macrophages are gated as single cells, viable and CD45^+^B220^-^CD3^-^Ly6G^-^F4/80^+^ CD11c^+^.

**Figure S17.**


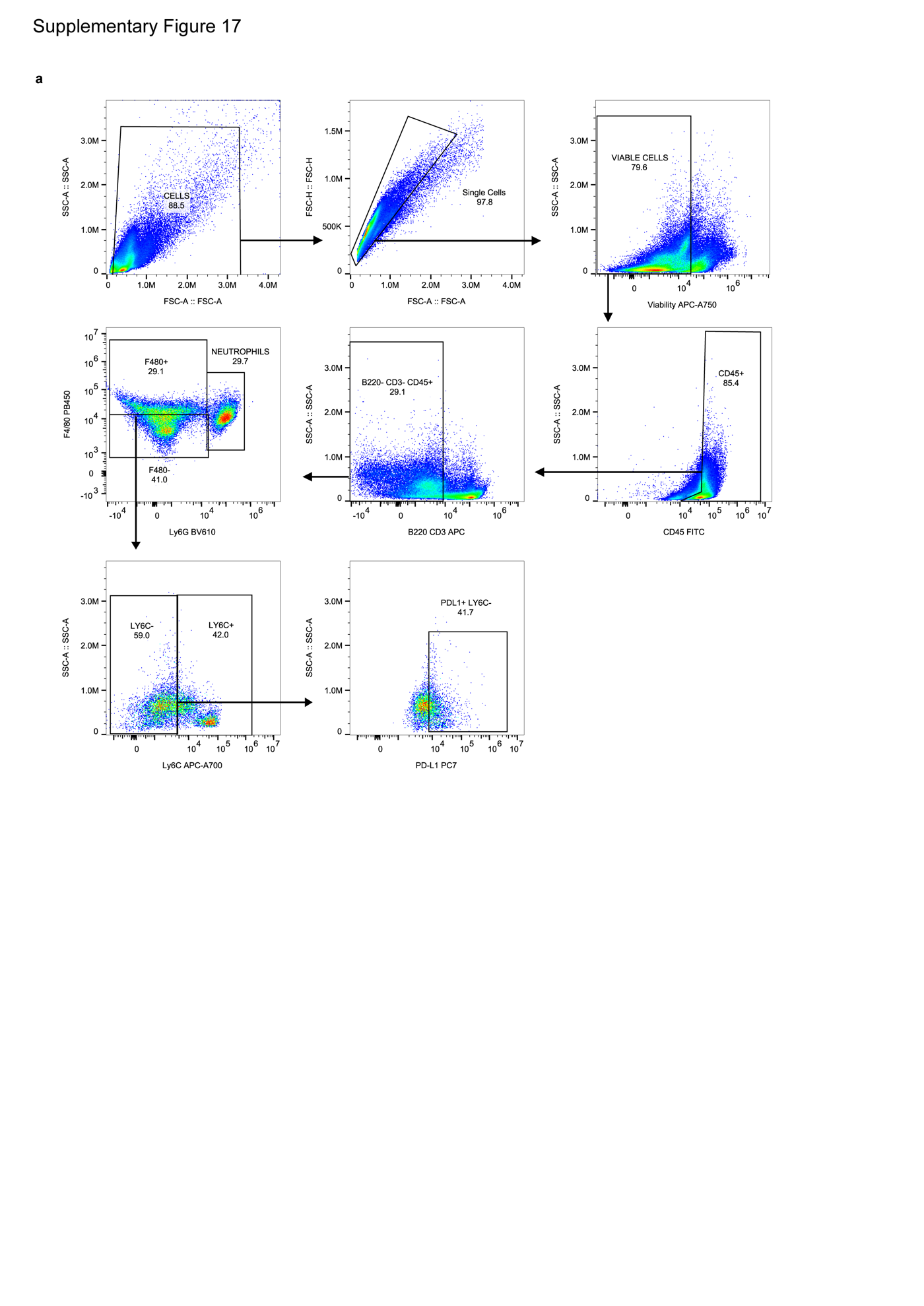


Supplementary Fig. 17. Gating strategy for PD-L1^+^ macrophages. Related to Figure 6. PD-L1^+^ macrophages are gated as single cells, viable and CD45^+^B220^-^CD3^-^Ly6G^-^F4/80^+^ Ly6C^-^PD-L1^+^.

**Figure S18.**


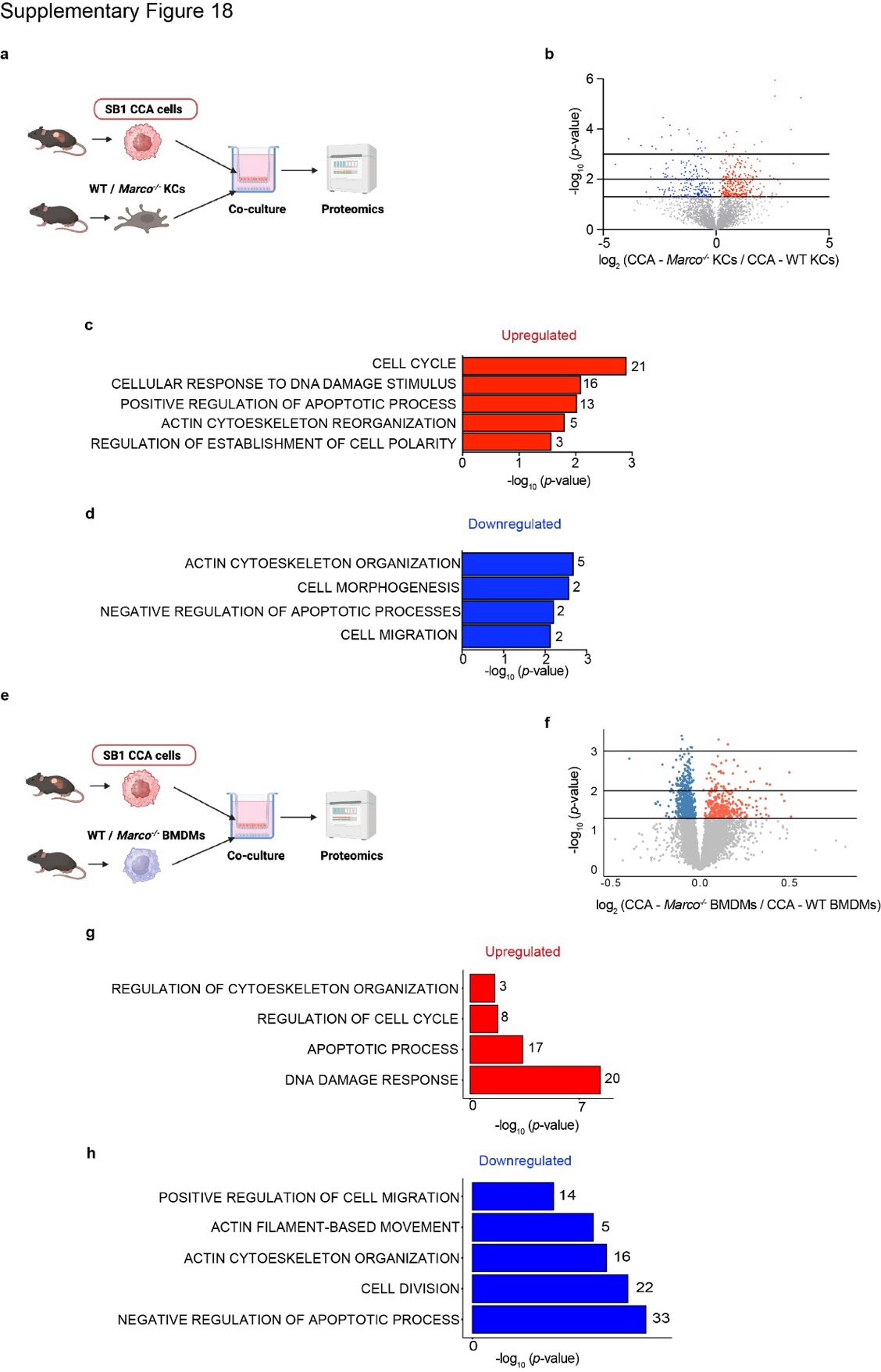


Supplementary Fig. 18. Proteomic analysis of mouse SB1 CCA cells co-cultured with WT or *Marco*^-/-^ KCs/BMDMs. (a) WT (n=4) and *Marco*^-/-^ (n=4) KCs were isolated and co-cultured with SB1 CCA cells for further proteomic analysis. (b) Volcano plot of all the identified proteins (n=3,158) by MS comparing the fold enrichment of these proteins in CCA cells co-cultured with *Marco*^-/-^ vs WT KCs. (c,d) GO analysis showing the (c) upregulated and (d) downregulated biological processes related to the dysregulated proteins in CCA cells co-cultured with *Marco*^-/-^ vs WT KCs. (e) WT (n=5) and *Marco*^-/-^ (n=5) BMDMs were isolated and co-cultured with SB1 CCA cells for further proteomic analysis. (f) Volcano plot of all the identified proteins (n=7,236) by MS comparing the fold enrichment of these proteins in CCA cells co-cultured with *Marco*^-/-^ vs WT BMDMs. (g,h) GO analysis showing the (g) upregulated and (h) downregulated biological processes related to the dysregulated proteins in CCA cells co-cultured with *Marco*^-/-^ vs WT BMDMs. Abbreviations: BMDM, Bone marrow-derived macrophage; CCA, cholangiocarcinoma; KC, Kupffer cell; GO, gene ontology; Marco, macrophage receptor with collagenous structure; MS, mass spectrometry; SB, sleeping beauty.

Table S1. Characteristics of patients with iCCA from the European CCA Histology Registry.

|  | ALL cases  (N=54) | MARCO^LOW^  (N=25) | MARCO^HIGH^  (N=29) | *p*-value |
| --- | --- | --- | --- | --- |
| Age (mean±SD) | 69±6.5 | 68±6.4 | 69±6.6 | ns |
| Gender | F: 22  M: 32 | F: 8  M: 17 | F: 14  M: 15 | ns |
| T  T1  T2  T3  T4 | 23  23  7  1 | 13  11  1  0 | 10  12  6  1 | ns |
| N  N0  N1 | 46  8 | 22  3 | 24  5 | ns |
| M  M0  M1 | 49  5 | 23  2 | 26  3 | ns |
| AJCC Stage  1  2  3  4 | 22  17  10  5 | 11  9  3  2 | 11  8  7  3 | ns |
| R  R0  R1  NA | 45  6  3 | 20  4  1 | 25  2  2 | ns |
| Growth Pattern  MF  PI  IG NA | 51  1  0  2 | 25  0  0  0 | 26  1  0  2 | ns |

Table S2. Antibodies and dyes used for flow cytometry.

| Antibody | Company | Reference |
| --- | --- | --- |
| APC anti-mouse CD45R (B220) | Tonbo | TO-20-0452 |
| BV510 anti-mouse CD45R | BioLegend | 103247; RRID: AB_2561394 |
| APC anti-mouse CD3 | BioLegend | 100312; RRID: AB_312677 |
| redFluor 710 anti-mouse CD3 | Tonbo | TO-80-0032 |
| BV605 anti-mouse CD4 | BioLegend | 100451; RRID: AB_2564591 |
| PerCP-Cy5.5 anti-mouse CD8a | Tonbo | TO-65-1886 |
| BV510 anti-mouse CD9 | BD Biosciences | 740128; RRID: AB_2739885 |
| BV510 anti-mouse CD11c | BioLegend | 117337; RRID: AB_2562010 |
| FITC anti-mouse CD45 | Tonbo | TO-35-0451 |
| PE-Cy7 anti-mouse CD45 | Tonbo | TO-60-0451 |
| AF700 anti-mouse CD90.2 (Thy1.2) | BioLegend | 105319; RRID: AB_493724 |
| PE-Cy7 anti-mouse CD127 | Tonbo | TO-60-1271 |
| PE-Cy7 anti-mouse CD279 (PD-1) | eBioscience | 25-9985-80; RRID: AB_10853672 |
| PE-Cy7 anti-mouse CD274 (PD-L1) | BioLegend | 124313; RRID: AB_10639934 |
| BV605 anti-mouse CD274 | BioLegend | 124321; RRID: AB_2563635 |
| PE anti-mouse CD152 (CTLA-4) | BioLegend | 106306; RRID: AB_313255 |
| BV421 anti-mouse F4/80 | BioLegend | 123131; RRID: AB_10901171 |
| AF700 anti-mouse Ly-6C | BioLegend | 128023; RRID: AB_10640119 |
| PE anti-mouse Ly-6C | BioLegend | 128007; RRID: AB_1186133 |
| BV605 anti-mouse Ly-6G | BioLegend | 127639; RRID: AB_2565880 |
| APC anti-mouse Ly-6G | Tonbo | TO-20-1276 |
| eFluor 450 anti-mouse NK1.1 | eBioscience | 48-5941-82; RRID: AB_2043877 |
| PE anti-mouse CD335 (NKp46) | eBioscience | 12-3351-80; RRID: AB_996682 |
| APC anti-mouse Lineage Antibody Cocktail | BD Biosciences | 558074; RRID: AB_1645213 |
| eFluor 780 Fixable Viability Dye | eBioscience | 65-0865-14 |

Abbreviations: AF, Alexa Fluor; APC, allophycocyanin; BV, Brilliant Violet; CD, cluster of differentiation; CTLA-4, cytotoxic T-lymphocyte associated protein 4; FITC, fluorescein isothiocyanate; Ly-6C, lymphocyte antigen 6 complex; Ly-6G, lymphocyte antigen 6 family member G; PE, phycoerythrin; PE-Cy, phycoerythrin-cyanine7 conjugate; PerCP, peridinin chlorophyll-A protein; PD-1, programmed cell death 1; PD-L1, programmed cell death-ligand 1; RRID, research resource identifiers.

**Table S3. Antibodies used for IHC and IF.**

| Antibody | Company | Reference | Species | Dilution |
| --- | --- | --- | --- | --- |
| Anti-CK19 | Abcam | ab84632 | Rabbit | 1:200 |
| Anti-MARCO | Atlas | HPA063793 | Rabbit | 1:300 |
| Anti-rabbit IgG biotinylated secondary antibody | Dako | E0353 | Swine | 1:200 |
| Anti-α-smooth muscle actin (α-SMA) | Dako | M0851 | Mouse | 1:100 |
| Anti-CD68 | Dako | M0876 | Mouse | 1:25 |
| Anti-CD206 | R&D | MAB25341 | Mouse | 1:25 |

Abbreviations: CK19, cytokeratin 19; IF, immunofluorescence; IgG, immunoglobulin G; IHC, immunohistochemistry; MARCO, Macrophage receptor with collagenous structure.

**Table S4. Mouse primers for qRT-PCR-based gene expression analysis.**

| Gene | Forward primer sequence (5´-3´) | Reverse primer sequence (5´-3´) |
| --- | --- | --- |
| *Marco* | TGATGCGACTGTCTTCTGTCG | CATTGTCCAGCCAGATGTTCC |
| *Il-10* | GCTCTTACTGACTGGCATGAG | CGCAGCTCTAGGAGCATGTG |
| *Tnf-α* | CCCTCACACTCAGATCATCTTCT | GCTACGACGTGGGCTACAG |
| *Col1a1* | TTCACCTACAGCACGCTTGTG | GATGACTGTCTTGCCCCAAGTT |
| *Acta2* | TCAGCGCCTCCAGTTCCT | AAAAAAAACCACGAGTAACAAATCAA |
| *Gapdh* | GCACAGTCAAGGCCGAGAAT | GCCTTCTCCATGGTGGTGAA |

Abbreviations: *Acta2*, actin alpha 2, smooth muscle; *Col1A1*, collagen type 1 alpha 1; *Gapdh*, glyceraldehyde 3-phosphate dehydrogenase; *Il*, interleukin; *Marco*, macrophage receptor with collagenous structure; *Tnf*, tumor necrosis factor.

**Table S5. Human primers for qRT-PCR-based gene expression analysis.**

| Gene | Forward primer sequence (5´-3´) | Reverse primer sequence (5´-3´) |
| --- | --- | --- |
| *MARCO* | CAGCGGGTAGACAACTTCACT | TTGTCTCCATCTCGTCCCATAG |
| *IL-6* | AAAGAGGCACTGGCAGAAAA | AGCTCTGGCTTGTTCCTCAC |
| *TNF-α* | CCTGCTGCACTTTGGAGTGA | CAGCTTGAGGGTTTGCTACA |
| *CD163* | TGGTCTTACTTCTCAGTGCC | TGAAAGTCCAGGAGGAGTG |
| *HPRT1* | TATGGCGACCCGCAGCCCT | CATCTCGAGCAAGACGTTCA |

Abbreviations: *CD163*, cluster of differentiation 163; *HPRT1*, hypoxanthine-guanine phosphoribosyltransferase; *IL*, interleukin; *MARCO*, macrophage receptor with collagenous structure; *TNF*, tumor necrosis factor.

Refereneces.

1. O’Rourke, C. J. *et al.* Identification of a Pan-Gamma-Secretase Inhibitor Response Signature for Notch-Driven Cholangiocarcinoma. *Hepatology* **71**, 196–213 (2020).

2. Andersen, J. B. *et al.* Genomic and genetic characterization of cholangiocarcinoma identifies therapeutic targets for tyrosine kinase inhibitors. *Gastroenterology* **142**, (2012).

3. Nakamura, H. *et al.* Genomic spectra of biliary tract cancer. *Nat. Genet.* **47**, 1003–10 (2015).

4. Job, S. *et al.* Identification of Four Immune Subtypes Characterized by Distinct Composition and Functions of Tumor Microenvironment in Intrahepatic Cholangiocarcinoma. *Hepatology* **72**, 965–981 (2020).

5. Jusakul, A. *et al.* Whole-Genome and Epigenomic Landscapes of Etiologically Distinct Subtypes of Cholangiocarcinoma. *Cancer Discov.* **7**, 1116–1135 (2017).

6. Dong, L. *et al.* Proteogenomic characterization identifies clinically relevant subgroups of intrahepatic cholangiocarcinoma. *Cancer Cell* **40**, 70-87.e15 (2022).

7. Chaisaingmongkol, J. *et al.* Common Molecular Subtypes Among Asian Hepatocellular Carcinoma and Cholangiocarcinoma. *Cancer Cell* **32**, 57--70.e3 (2017).

8. Ramachandran, P. *et al.* Resolving the fibrotic niche of human liver cirrhosis at single-cell level. *Nature* **575**, 512–518 (2019).

9. Ma, L. *et al.* Tumor Cell Biodiversity Drives Microenvironmental Reprogramming in Liver Cancer. *Cancer Cell* **36**, 418--430.e6 (2019).

10. Zhang, M. *et al.* Single-cell transcriptomic architecture and intercellular crosstalk of human intrahepatic cholangiocarcinoma. *J. Hepatol.* **73**, 1118–1130 (2020).

11. Ma, L. *et al.* Single-cell atlas of tumor cell evolution in response to therapy in hepatocellular carcinoma and intrahepatic cholangiocarcinoma. *J. Hepatol.* **75**, 1397–1408 (2021).

12. Jimenez-Sanchez, A., Cast, O. & Miller, M. L. Comprehensive Benchmarking and Integration of Tumor Microenvironment Cell Estimation Methods. *Cancer Res.* **79**, 6238–6246 (2019).

13. Jiang, P. *et al.* Signatures of T cell dysfunction and exclusion predict cancer immunotherapy response. *Nat. Med.* **24**, 1550–1558 (2018).

14. Xu, L. *et al.* TIP: A Web Server for Resolving Tumor Immunophenotype Profiling. *Cancer Res.* **78**, 6575–6580 (2018).

15. Yamada, D. *et al.* IL-33 facilitates oncogene-induced cholangiocarcinoma in mice by an interleukin-6-sensitive mechanism. *Hepatology* **61**, 1627–42 (2015).

16. Wang, J. *et al.* Loss of Fbxw7 synergizes with activated Akt signaling to promote c-Myc dependent cholangiocarcinogenesis. *J. Hepatol.* **71**, 742–752 (2019).

17. Loeuillard, E. *et al.* Targeting tumor-associated macrophages and granulocytic myeloid-derived suppressor cells augments PD-1 blockade in cholangiocarcinoma. *J. Clin. Invest.* **130**, 5380–5396 (2020).

18. Masetti, M. *et al.* Lipid-loaded tumor-associated macrophages sustain tumor growth and invasiveness in prostate cancer. *J. Exp. Med.* **219**, (2022).

19. Perugorria, M. J. *et al.* Tumor progression locus 2/Cot is required for activation of extracellular regulated kinase in liver injury and toll-like receptor-induced TIMP-1 gene transcription in hepatic stellate cells in mice. *Hepatology* **57**, 1238–1249 (2013).

20. Uriarte, I. *et al.* Bicarbonate secretion of mouse cholangiocytes involves Na(+)-HCO(3)(-) cotransport in addition to Na(+)-independent Cl(-)/HCO(3)(-) exchange. *Hepatology* **51**, 891–902 (2010).

21. Wiśniewski, J. R., Zougman, A., Nagaraj, N. & Mann, M. Universal sample preparation method for proteome analysis. *Nat. Methods* **6**, 359–62 (2009).

22. Meier, F. *et al.* Parallel Accumulation-Serial Fragmentation (PASEF): Multiplying Sequencing Speed and Sensitivity by Synchronized Scans in a Trapped Ion Mobility Device. *J. Proteome Res.* **14**, 5378–87 (2015).

23. Meier, F. *et al.* Online Parallel Accumulation-Serial Fragmentation (PASEF) with a Novel Trapped Ion Mobility Mass Spectrometer. *Mol. Cell. Proteomics* **17**, 2534–2545 (2018).

24. Babicki, S. *et al.* Heatmapper: web-enabled heat mapping for all. *Nucleic Acids Res.* **44**, W147-53 (2016).

25. Huang, D. W., Sherman, B. T. & Lempicki, R. A. Systematic and integrative analysis of large gene lists using DAVID bioinformatics resources. *Nat. Protoc.* **4**, 44–57 (2009).

**^§^European CCA Histology Registry**

**Members:** Ana Lleo^1,2^, Rocio IR Macias^3^, Vincenzo Cardinale^4^, Stefano Leone^5^, Ludovica Ceci^5^, Gian Luca Grazi^6^, Laura Izquierdo-Sanchez^7,8^, Pilar Acedo^9^, Michail Doukas^10^, Lara R Heij^10,11,12,13^, Konrad Reichel^13^, Paolo Onori^5^, Barbara Franceschini^14^, Luca Di Tommaso^1,15^, Pedro M Rodrigues^7,8,16^, Guido Torzilli^1,17^, Diego Bueno-Sacristan^18^, Andres Garcia-Sampedro^9,19^, Alberto Quaglia^20^, Francesco Ardito^21^, Felice Giuliante^21^, Bas Groot Koerkamp^22^, Stephen P Pereira^9^, Domenico Alvaro^4^, Jesus M Banales^7,8,16,23^, Diletta Overi^5^, Eugenio Gaudio^5*^, Guido Carpino^5*^.

**Affiliations**

^1^Department of Biomedical Sciences, Humanitas University, Milan, Italy. ^2^Division of Internal Medicine and Hepatology, Department of Gastroenterology, IRCCS Humanitas Research Hospital, Rozzano, Milan, Italy. ^3^Laboratory of Experimental Hepatology and Drug Targeting (HEVEPHARM), National Institute for the Study of Liver and Gastrointestinal Diseases (CIBERehd), IBSAL, University of Salamanca, Salamanca, Spain. ^4^Department of Translational and Precision Medicine, Sapienza University of Rome, Rome, Italy. ^5^Department of Anatomical, Histological, Forensic Medicine and Orthopaedic Sciences, Sapienza University of Rome, Rome, Italy. ^6^Department of Experimental and Clinical Medicine, HepatoBiliaryPancreatic Surgery, University of Florence, Florence, Italy. ^7^Department of Liver and Gastrointestinal Diseases, Biogipuzkoa Health Research Institute, Donostia University Hospital, University of the Basque Country (UPV/EHU), San Sebastian, Spain. ^8^National Institute for the Study of Liver and Gastrointestinal Diseases (CIBERehd), Carlos III National Health Institute, Madrid, Spain. ^9^Institute for Liver and Digestive Health, University College London, London, UK. ^10^Department of Pathology and Clinical Bioinformatics, Erasmus University Medical Centre, Rotterdam, the Netherlands. ^11^Department of Pathology, University Hospital Essen, Essen, Germany. ^12^Department of Renal and Hypertensive Disorders, Rheumatological and Immunological Diseases (Medical Clinic II), Medical Faculty, RWTH Aachen University, Aachen, Germany. ^13^Department of Surgery and Transplantation, University Hospital Essen, Essen, Germany. ^14^Laboratory of Hepatobiliary Immunopathology, IRCCS Humanitas Research Hospital, Rozzano, Milan, Italy. ^15^Pathology Unit, IRCCS Humanitas Research Hospital, Rozzano, Milan, Italy. ^16^Ikerbasque, Basque Foundation for Science, Bilbao, Spain. ^17^Division of Hepatobiliary and General Surgery, Department of Surgery, IRCCS Humanitas Research Hospital, Rozzano, Milan, Italy. ^18^Servicio de Patologia, Hospital Universitario de Salamanca, IBSAL, Salamanca, Spain. ^19^Department of Chemical Engineering and Biotechnology, University of Cambridge, Cambridge, UK. ^20^Department of Cellular Pathology, UCL Cancer Institute, Royal Free Hospital, London, United Kingdom. ^21^Hepatobiliary Surgery Unit, Foundation and Teaching Hospital Sacred Heart IRCCS A. Gemelli, Catholic University of the Sacred Heart, Rome, Italy. ^22^Department of Surgery, Erasmus MC Cancer Institute, Rotterdam, the Netherlands. ^23^Department of Biochemistry and Genetics, School of Sciences, University of Navarra, Pamplona, Spain. *coordinators of the Registry
